# Mutations in the *ilvN* gene mitigate growth inhibitory effect of cysteine in *Corynebacterium glutamicum*

**DOI:** 10.1007/s00253-025-13444-y

**Published:** 2025-03-10

**Authors:** Kazuho Matsuhisa, Katsuhiro Ogawa, Kento Komata, Takashi Hirasawa

**Affiliations:** School of Life Science and Technology, Institute of Science Tokyo, 4259 Nagatsuta-cho, Midori-Ku, Yokohama, Kanagawa 226-8501 Japan

**Keywords:** *Corynebacterium glutamicum*, Adaptive laboratory evolution, Cysteine, Growth inhibition, *ilvN*, Valine

## Abstract

**Abstract:**

Cysteine, a common amino acid used in food, cosmetic, and pharmaceutical industries, has a growth inhibitory effect. This growth inhibition by cysteine poses a problem, as the production of cysteine using microbial cells results in decreased cell growth and cysteine productivity. The underlying mechanism of growth inhibition by cysteine is unclear. This study aims to understand the mechanism of growth inhibition by cysteine in *Corynebacterium glutamicum*. To do this, cysteine-resistant mutants of *C. glutamicum* were isolated based on adaptive laboratory evolution (ALE) and their characteristics were analyzed. Genome resequencing revealed that mutations in the open reading frame of the *ilvN* gene encoding the regulatory small subunit of acetohydroxyacid synthase (AHAS), which is involved in branched-chain amino acid biosynthesis, were found in ALE cell populations and the isolated cysteine-resistant mutants. The *ilvN* mutations which are responsible for increased valine production resulted in improved cell growth in the presence of cysteine. Moreover, the addition of valine to the culture medium mitigated growth inhibition by cysteine, whereas the addition of leucine and isoleucine showed a slight mitigation. Additionally, the activity of AHAS from *C. glutamicum* was inhibited by cysteine, whereas AHAS from the strains carrying *ilvN* mutations exhibited resistance to cysteine. These results indicate that growth inhibition by cysteine is caused by perturbations in the biosynthesis of branched-chain amino acids, particularly valine in *C. glutamicum*. Furthermore, the cysteine-resistant mutants obtained by ALE demonstrated enhanced cysteine production as production hosts, suggesting that cysteine resistance is a useful phenotype for cysteine production in *C. glutamicum*.

**Key points:**

• *Cysteine-resistant mutants of C. glutamicum obtained by ALE were analyzed.*

• *Perturbation of valine biosynthesis by cysteine results in growth inhibition in C. glutamicum.*

• *Cysteine resistance is a useful phenotype for cysteine production by C. glutamicum.*

**Supplementary Information:**

The online version contains supplementary material available at 10.1007/s00253-025-13444-y.

## Introduction

Cysteine is a sulfur-containing amino acid utilized in food, cosmetic, and pharmaceutical industries because of its antioxidative effect. However, cysteine is known as an amino acid exhibiting growth inhibitory effect. The studies on the effect of cysteine on growth in microbial cells caused by inhibition of amino acid biosynthesis had been reported after the 1960s (Adiga et al. 1962; Kovács et al. 1968; Kari et al. 1971; Harris 1981; Cowman et al. 1983). Moreover, the studies on growth inhibition by cysteine related to oxidative stress had also been reported (Bhuvaneswaran et al. 1964; Carlsson et al. 1979; Gomez et al. 1980; Berglin et al. 1982). In addition, Park and Imlay (2003) showed by the analysis using *Escherichia coli* cells that cysteine reduced Fe^3+^ to Fe^2+^, which reacts with hydrogen peroxide (H_2_O_2_) to produce hydroxyl radical (•OH) and hydroxide ion (OH^–^); this reaction is called Fenton reaction. The Fenton reaction is related to hydroxyl radical production:

$${\mathrm{Fe}}^{2+}+{\mathrm{H}}_{2}{\mathrm{O}}_{2}\to {\mathrm{Fe}}^{3+}+ \bullet \mathrm{OH}$$

Hydroxyl radical is a cause of oxidative stress, inducing damage to high-molecular-weight cellular components such as DNA and proteins, and this phenomenon results in a decrease in cell growth.

Cysteine has industrially been produced from the hydrolysis of proteins and animal hairs, but that process has some problems, such as low productivity, unpleasant odors, and wastewater treatment. Therefore, cysteine production processes using microbial cells have been developed as one of the alternative production processes. Recently, cysteine production by *E. coli* and *Pantoea ananatis* has been reported (Kawano et al. 2015, 2017; Takumi et al. 2017). Moreover, the studies on cysteine production by metabolically engineered *Corynebacterium glutamicum* have been reported as well (Kishino et al. 2019; Kondoh and Hirasawa 2019; Wei et al. 2019); *C. glutamicum* is known as a glutamic acid-producing bacterium (Kinoshita et al. 1957) and is utilized for production of various chemicals including amino acids (Sano and Shiio 1970; Shiio and Nakamori 1970), organic acids (Okino et al. 2005), and proteins (Kikuchi et al. 2003).

Growth inhibition by cysteine in microbial cells poses a significant challenge in the bioproduction of cysteine and its derivatives because the high concentration of cysteine reduces the growth of host microorganisms as well as the productivity of target chemicals. Therefore, understanding the mechanism of growth inhibitory effect of cysteine is crucial for acquiring cysteine resistance to host microbial cells toward the bioproduction of cysteine and its derivatives. In order to investigate and understand the mechanism of growth inhibition by cysteine in *C. glutamicum*, we isolated cysteine-resistant mutants of *C. glutamicum* by adaptive laboratory evolution (ALE) based on repeated serial transfers of culture in the presence of cysteine. Moreover, the mutations related to cysteine resistance were identified by whole genome resequencing of the cysteine-resistant mutants obtained via ALE. Furthermore, we evaluated the effectiveness of the cysteine resistance on cysteine production by *C. glutamicum*.

## Materials and methods

### Strains and media

*C. glutamicum* wild-type strain NBRC 12168 (synonymous with the type strain ATCC 13032), which was obtained from the Biological Resource Center, National Institute of Technology and Evaluation (Kisarazu, Chiba, Japan), and its *aecD* gene disruptant, which was constructed in the previous study (Kondoh and Hirasawa 2019), were used in this study. *E. coli* JM109 (*recA*1 *endA*1 *gyrA*96 *thi hsdR*17(r_K_^–^ m_K_^+^) e14^–^(*mcrA*^–^) *supE*44 *relA*1 Δ(*lac-proAB*)/F´(*traD*36 *proAB*^+^ *lacI*^q^ *lacZ*ΔM15)) was used for the construction of plasmids.

In recombinant DNA experiments and cysteine production assays of *C. glutamicum*, Lennox (L) medium (1% hipolypepton (Shiotani M.S. Co., Ltd., Hyogo, Japan), 0.5% dried yeast extract D-3H (Shiotani M.S. Co., Ltd.), 0.5% NaCl, and 0.1% glucose; pH 7.0) was used. For ALE of *C. glutamicum* and evaluation of cysteine resistance in *C. glutamicum*, modified M9 medium (11.28 g L^−1^ Difco M9 minimal salts, 5 × (Becton, Dickinson and Company, Franklin Lakes, NJ), 4 g L^−1^ glucose, 2 mM MgSO_4_·7H_2_O, 0.1 mM CaCl_2_·2H_2_O, 0.02 mM FeSO_4_·7H_2_O, 200 µg L^−1^ biotin, 1 mg L^−1^ thiamine·HCl) was used. In the cysteine production assay, a semisynthetic medium (80 g L^−1^ glucose, 30 g L^−1^ (NH_4_)_2_SO_4_, 3.0 g L^−1^ Na_2_HPO_4_·12H_2_O, 6.0 g L^−1^ KH_2_PO_4_, 2.0 g L^−1^ NaCl, 84 mg L^−1^ CaCl_2_, 3.9 mg L^−1^ FeCl_3_, 0.9 mg L^−1^ ZnSO_4_·7H_2_O, 0.3 mg L^−1^ CuCl_2_·2H_2_O, 5.56 mg L^−1^ MnSO_4_·5H_2_O, 0.1 mg L^−1^ (NH_4_)_6_Mo_7_O_24_·4H_2_O, 0.3 mg L^−1^ Na_2_B_4_O_7_·10H_2_O, 0.4 g L^−1^ MgSO_4_·7H_2_O, 40 mg L^−1^ FeSO_4_·7H_2_O, 0.5 mg L^–1^ thiamine·HCl, 0.1 g L^−1^ ethylenediamine-*N*, *N*, *N*´, *N*´-tetraacetic acid disodium salt dihydrate, 0.02 mg L^−1^ D-biotin, 10 g L^−1^ dried yeast extract D-3H, and 25 g L^−1^ CaCO_3_; pH 7.2) (Kondoh and Hirasawa 2019) was used as a main production medium. To prepare the agar plate media, 1.5% agar was added. When necessary, 10 and 20 mg L^−1^ kanamycin was added to the medium for *C. glutamicum* and *E. coli*, respectively.

### ALE of *C. glutamicum* to obtain cysteine-resistant mutants

Initially, the *aecD* gene disruptant of *C. glutamicum* was cultured in a modified M9 medium containing 1.5 g L^−1^ L-cysteine hydrochloride monohydrate (Cys·HCl·H_2_O) for 24 h. The culture was then diluted with the fresh modified M9 medium containing the same concentration of Cys·HCl·H_2_O to achieve the optical density at 660 nm (OD_660_) of 0.1, and the cells were then cultured for 24 h; OD_660_ values were measured using a spectrophotometer UVmini-1240 (Shimadzu Corporation, Kyoto, Japan). The serial transfer process was repeated until the specific growth rate increased. After that, the concentration of Cys·HCl·H_2_O in the M9 medium was increased from 1.5 to 2 g L^−1^ and the serial transfer process was repeated. The Cys·HCl·H_2_O concentration was stepwise increased to 2.5, 3, and 4 g L^−1^. After the serial transfer, the previous culture was mixed with glycerol (15% (v/v), final concentration) and then stored at − 80 °C. Finally, the ALE experiments were continued until the specific growth rate of the cultured cells increased and became stable in the presence of 4 g L^−1^ Cys·HCl·H_2_O. In the present study, five series of cultures for ALE, designated ALE1, ALE2, ALE3, ALE4, and ALE5, were independently performed.

### Isolation of cysteine-resistant mutants from ALE cultures

The cultures obtained at the end of the ALE were spread on L agar plates and incubated at 30 °C for 3 days. Ten colonies on the plates were randomly picked and cultured in L liquid medium followed by making frozen stocks for each colony. Some aliquots of the prepared frozen stocks were transferred to the L liquid medium and cultured at 30 °C overnight. The culture was then diluted with a modified M9 medium containing 4 g L^−1^ Cys·HCl·H_2_O to achieve OD_660_ of 0.1–0.15. The diluted culture was incubated at 30 °C for 24 h and then diluted again with modified M9 medium containing 4 g L^−1^ Cys·HCl·H_2_O to achieve OD_660_ of 0.1. The diluted culture was incubated at 30 °C, and time courses of OD_660_ were measured during incubation. Among the ten strains obtained from a single ALE series, four or five strains exhibiting the highest specific growth rate in the presence of 4 g L^−1^ Cys·HCl·H_2_O were selected and cultured again in the presence of the same concentration of Cys·HCl·H_2_O followed by the selection of two strains showing the highest specific growth rates.

Next, the frozen stocks of the two selected strains were spread on L agar plates. After colonies appeared on the L agar plates, the same culture procedure was conducted on the ten randomly chosen colonies, and the strains exhibiting the highest specific growth rate in the presence of 4 g L^−1^ Cys·HCl·H_2_O were obtained as cysteine-resistant mutants.

### Whole genome resequencing

For whole genome resequencing, genomic DNA from the ALE cultures and isolated cysteine-resistant mutants was extracted using Gentra Puregene Yeast/Bact. Kit (Qiagen N.V., Venlo, Netherland). The whole genome resequencing analysis including library preparation, sequence runs, and data analysis was conducted by Eurofins Genomics K.K. (Tokyo, Japan).

### Introduction of *ilvN* mutations into the *C. glutamicum* genome

The mutant *ilvN* genes were amplified from the genome DNA of the cell populations obtained via ALE and cysteine-resistant mutants by polymerase chain reaction (PCR) using KOD One PCR Master Mix (Toyobo Co., Ltd., Osaka, Japan) and primer set 5′-CACGAATTCATGACCAACCAGGAACTCAC-3′ and 5′-ATGGGATCCAGTTTAACTCGCTTGGGCAG-3′ and were cloned into the EcoRI-BamHI sites of the pK18*mobsacB* (Schäfer et al. 1994). The resulting plasmids were introduced into the *aecD* gene disruptant of *C. glutamicum*. In kanamycin-resistant transformants, the plasmid should be inserted into the *ilvN* locus on the genome by homologous recombination because the plasmid cannot replicate in *C. glutamicum* cells. The kanamycin-resistant transformants were cultured in liquid L medium to induce pop-out of the pK18*mobsacB* sequence, and sucrose-resistant colonies were obtained by spreading the culture onto L agar plates containing 20% sucrose. If the pK18*mobsacB* sequence is removed from the genome of kanamycin-resistant transformants by homologous recombination, the cells exhibit sucrose resistance because pK18*mobsacB* carries the *sacB* gene, the product of which is responsible for sucrose sensitivity in *C. glutamicum*. To confirm the successful introduction of mutations into the *ilvN* locus, *ilvN* locus was amplified by PCR from the genome of sucrose-resistant cells using the same primer sets for amplifying *ilvN* gene described above, and the sequence of the amplified fragments was checked.

### Analysis of the *katA* gene expression by reverse transcription-quantitative PCR

For total RNA extraction, cells of the *aecD* gene disruptant cultured in modified M9 medium with and without the addition of 0.1 g L^−1^ valine for 8 h were harvested by centrifugation and stored at − 80 °C until RNA extraction. In addition, the *ilvN* mutation-carrying *aecD* gene disruptant culture in a modified M9 medium for 8 h was harvested. Total RNA was extracted from frozen cells using nucleospin RNA (Macherey–Nagel GmbH & Co. KG, Düren, Germany). Reverse transcription (RT) was performed using the ReverTra Ace qPCR RT Master Mix with gDNA Remover (Toyobo Co., Ltd., Osaka, Japan). Quantitative PCR (qPCR) was conducted using Thunderbird Next SYBR qPCR Mix (Toyobo Co., Ltd.) and a Thermal Cycler Dice Real Time System III (Takara Bio Inc., Shiga, Japan). Expression of the *katA* gene in the presence of valine relative to that in the absence of valine was determined by the ΔΔCt method and normalized by the expression of the 16S rRNA gene as a housekeeping gene. Expression of the *katA* gene in the *ilvN* mutation-carrying *aecD* gene disruptant relative to that in the *aecD* gene disruptant was analyzed as well. The primer sets used for qPCR were 5′-GCAGAGAACTACCGCTGGAA-3′ and 5′-AAGATACGTGCCTGGAGCAT-3′ for *katA* and 5′-CTTACCTGGGCTTGACATGG-3′ and 5′-CACCATAATGTGCTGGCAAC-3′ for 16S rRNA.

### Disruption of the *katA* gene in *C. glutamicum*

The upstream and downstream regions of the *katA* gene open reading frame (ORF) were amplified by PCR from the *C. glutamicum* NBRC 12168 genome, using primer sets 5′-TCTGAATTCGTATTCGACGATGGATTTGC-3′ and 5′-CCTTGACGCGCTCAGACATTAGCATTCCTTCC-3′ for the upstream region and 5′-AATGTCTGAGCGCGTCAAGGAGCTTTACCTCC-3′ and 5′-TGGAAGCTTCCAGTCACATTCAAACCGTG-3′ for the downstream region. Then, the amplified fragments were connected into a single fragment by overlap extension PCR. The connected fragment was cloned into the EcoRI-HindIII sites of pK18*mobsacB*. The resulting plasmid was introduced into the *aecD* gene disruptant of *C. glutamicum* to obtain kanamycin-resistant transformants. In kanamycin-resistant transformants, the constructed plasmid should be inserted upstream or downstream of the *katA* ORF by homologous recombination. The kanamycin-resistant transformants were cultured in liquid L medium to induce pop-out of the pK18*mobsacB* sequence together with the *katA* ORF by homologous recombination, and sucrose-resistant colonies were obtained by spreading the culture onto L agar plates containing 20% sucrose. The disruption of *katA* gene was confirmed by PCR from the genome of sucrose-resistant cells using a primer set, 5′-TCTGAATTCGTATTCGACGATGGATTTGC-3′ and 5′-TGGAAGCTTCCAGTCACATTCAAACCGTG-3′.

### Analysis of cysteine resistance of *C. glutamicum*

Cells of *C. glutamicum* grown in L medium were harvested by centrifugation and suspended in a modified M9 medium. Then, the cells were inoculated into M9 medium containing Cys·HCl·H_2_O to achieve the OD_660_ of 0.1 and cultured at 30 °C. For the analysis of cysteine resistance in the isolated cysteine-resistant mutants, cells were cultured in 5 mL of medium with reciprocal shaking at 150 strokes min^−1^. During cultivation, OD_660_ was manually measured using a spectrophotometer.

For evaluating cysteine resistance of the recombinant strains carrying *ilvN* mutations and *katA* disruptant and the effect of branched amino acids on growth of the *aecD* gene disruptant in the presence of cysteine, cells were cultured in 4 mL of M9 medium containing Cys·HCl·H_2_O and branched-chain amino acids with shaking at 40 rpm using a compact rocking incubator TVS062CA (Advantec Toyo Kaisha, Ltd., Tokyo, Japan), in which OD_660_ can be measured automatically.

### Measurement of concentration of amino acids in culture supernatants

The concentration of branched-chain amino acids in the culture supernatants of *C. glutamicum* obtained by centrifugation of the cultures was determined using a high-performance liquid chromatography system, Nexera lite (Shimadzu Co., Ltd.), equipped with a refractive index detector, RID-20A (Shimadzu Co., Ltd.), and a cation chromatography column, Shodex IC YS-50 (Resonac Corporation, Tokyo, Japan). Phosphoric acid solution (6 mM) was used as the mobile phase. The column temperature and flow rate of the mobile phase were set at 40 °C and 1 mL min^−1^, respectively.

Analyses of other amino acids in culture supernatants with high detection sensitivity were conducted using the Nexera X2 precolumn amino acid analysis system (Shimadzu Co., Ltd.). Amino acid standard purchased from Sigma-Aldrich Co. LLC (St. Louis, MO) was used for quantification.

### Measurement of acetohydroxyacid synthase activity

Activities of acetohydroxyacid synthase (AHAS) in the *aecD* disruptant and *ilvN* mutation-carrying *aecD* gene disruptant of *C. glutamicum* were determined based on the conversion of pyruvate to acetolactate by the methods reported by Leyval et al. (2003). Cells were cultured in 50 mL of M9 medium in a 300-mL baffled flask at 30 °C for 8 h with a rotary shaking at 200 rpm and then harvested by centrifugation at 17,000 × *g* at 4 °C for 15 min. The harvested cells washed two times with 2% KCl solution were resuspended in the disruption buffer, which contains 0.5 mM dithiothreitol and 20% glycerol in 100 mM potassium phosphate buffer (pH 7.3), and stored at − 30 °C until measurement.

To obtain crude extracts, cells suspended in the disruption buffer were disrupted by sonication. Cell debris was removed by centrifugation at 12,000 × *g* at 4 °C for 15 min and the supernatant was kept on ice. Protein concentration in the crude extracts was determined using Bio-Rad protein assay dye reagent concentrate (Bio-Rad Laboratories Inc., Hercules, CA).

For AHAS activity measurement, 900 µL of reaction mixture containing 50 mM sodium pyruvate, 10 mM MgCl_2_, 100 µM thiamine pyrophosphate, and 100 µM FAD in 100 mM potassium phosphate buffer (pH 7.4) was prepared. When necessary, 1 mM valine or 0.1 g L^−1^ Cys·HCl·H_2_O was added to the reaction mixture. The enzyme reaction was started by adding 100 µL of crude extracts containing 0.1 g L^−1^ protein to the reaction mixture, and the mixture was incubated at 37 °C for 90 min. The reaction was terminated by adding 100 µL of 50% H_2_SO_4_ solution followed by incubation at 37 °C for 25 min; in this process, acetolactate formed from pyruvate by AHAS-catalyzed reaction was converted to acetoin. Acetoin formation was determined by the methods reported by Yang et al. (2000). In this study, AHAS activities in the presence of valine or cysteine relative to that in the absence of them were calculated.

### Cysteine production by engineered cysteine-resistant mutants

For engineering the cysteine-resistant mutants obtained by ALE to construct cysteine-producing strains, the plasmid pCYS*_Cg(T94A)-SER* carrying the mutant *cysE* and *serA* genes from *C. glutamicum* (Kondoh and Hirasawa 2019), whose products are resistant to feedback inhibition by cysteine and serine, respectively, was introduced into the cysteine-resistant mutants. In the cysteine production assay, 1.6 mL culture of the engineered cysteine-resistant mutants grown in L liquid medium was transferred to 300-mL baffled flasks containing 40 mL of semisynthetic medium and cultured at 30 °C with rotary shaking at 200 rpm. During cultivation, cell growth was monitored by measuring the OD_660_ after dilution of the culture with 0.2 N HCl to dissolve CaCO_3_ in the culture, and the culture supernatant was obtained by centrifugation. The cysteine concentration in the culture supernatant was measured as described by Kondoh and Hirasawa (2019).

## Results

### Effect of cysteine on the growth of *C. glutamicum*

**Fig. 1:**
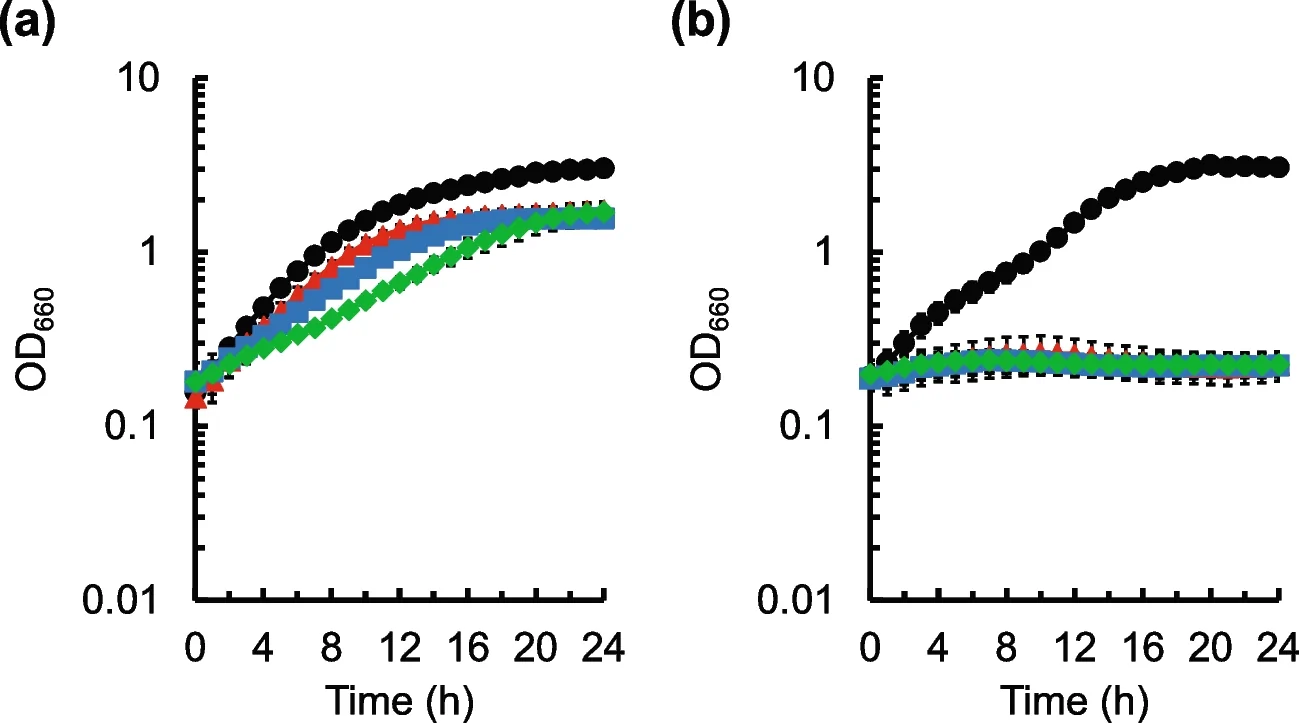
Effect of cysteine on the growth of *C. glutamicum*. Cell growth of the *C. glutamicum* NBRC 12168 (**a**) and its *aecD* gene disruptant (**b**) in modified M9 medium without cysteine addition (black circles) and with the addition of 0.25 (red triangles), 0.5 (blue squares), and 1 (green diamonds) g L^−1^ of Cys·HCl·H_2_O is shown. The average ± standard deviation of the data in three independent cultures is shown

Firstly, the effect of cysteine on the growth of the *C. glutamicum* wild-type NBRC 12168 in the modified M9 medium was analyzed (Fig. 1a). In this study, the medium that does not contain natural components such as peptone and yeast extracts was used to evaluate the effect of cysteine on cell growth because the effect of unknown nutrients in natural components needs to be avoided. In the wild-type strain, cysteine inhibited its growth and the degree of growth inhibition was dose-dependent when adding 0.25–1 g L^−1^ Cys·HCl·H_2_O to the medium; the specific growth rate during exponential growth decreased as the cysteine concentration added to the medium increased (Supplementary Table S1). However, cell growth was not completely inhibited by the addition of 0.5–2 g L^−1^ Cys·HCl·H_2_O.

Next, the effect of cysteine on the growth of the disruptant of the *aecD* gene encoding cysteine desulfhydrase, which is responsible for cysteine degradation, was analyzed (Fig. 1b). The growth of the *aecD* disruptant was dramatically reduced even in the presence of 0.25 g L^−1^ Cys·HCl·H_2_O (Fig. 1b). Similar to the result in the NBRC 12168, the specific growth rates reduced as the cysteine concentration added to the medium increased (Supplementary Table S1). The results indicate that the *aecD* disruptant exhibits more sensitivity to cysteine than the wild-type strain because the *aecD* disruptant cannot degrade cysteine incorporated into cells.

### ALE of *C. glutamicum* in the presence of cysteine

In order to obtain cysteine-resistant mutants of *C. glutamicum*, ALE in the presence of cysteine was performed. In the present study, the *aecD* gene disruptant was used to avoid degrading cysteine by the cells during ALE. Here, five independent ALE series, namely ALE1, ALE2, ALE3, ALE4, and ALE5, were conducted. The growth profiles of five ALE experiments are shown in Supplementary Fig. S1. Cell growth in all five ALE series was finally observed in a modified M9 medium containing 4 g L^−1^ Cys·HCl·H_2_O after 2200–2300 h, which corresponds to 330–370 generations, and the specific growth rate reached approximately 0.13 h^−1^.

To confirm whether the cultures obtained at the end of the ALE actually contain cells that can grow in the presence of cysteine, growth of the cell populations at the end of the ALE in the medium containing 4 g L^−1^ Cys·HCl·H_2_O was measured. As shown in Supplementary Fig. S2, cell populations in all ALE cultures could grow well in the presence of 4 g L^−1^ Cys·HCl·H_2_O. This result indicates that cell populations in the ALE cultures contain mutants that can grow in the presence of 4 g L^−1^ Cys·HCl·H_2_O.

Furthermore, to confirm whether the cell populations in the ALE cultures stably exhibited cysteine resistance, serial transfer of the ALE cultures to the modified M9 medium without cysteine addition was repeated five times. The cultures were then transferred to the same medium containing 4 g L^−1^ Cys·HCl·H_2_O, and the growth was measured. As shown in Supplementary Fig. S3, cell populations of two ALE culture lineages, ALE2 and ALE5, showed higher growth in the presence of 4 g L^−1^ Cys·HCl·H_2_O compared to those of the other ALE culture linages, ALE1, ALE3, and ALE4. Therefore, ALE2 and ALE5 were chosen to isolate cysteine-resistant mutants of *C. glutamicum* because it was expected that the genomes of the cells in these ALE cultures have mutations related to cysteine resistance.

### Isolation of cysteine-resistant mutants of *C. glutamicum* from ALE cultures

The cysteine-resistant mutants of *C. glutamicum* were obtained from the cultures of ALE2 and ALE5 obtained at the end of ALE experiments (2279 h; 362 and 352 generations, respectively) as described in the “Materials and methods” section. Among the ten strains, four and five strains from ALE2 and ALE5, respectively, showing the highest specific growth rates in the presence of 4 g L^−1^ Cys·HCl·H_2_O, namely ALE2_1 (0.12 h^−1^), ALE2_2 (0.13 h^−1^), ALE2_4 (0.14 h^−1^), and ALE2_10 (0.12 h^−1^) from ALE2 and ALE5_2 (0.14 h^−1^), ALE5_6 (0.12 h^−1^), ALE5_7 (0.15 h^−1^), ALE5_9 (0.12 h^−1^), and ALE5_10 (0.12 h^−1^) from ALE5, were selected and cultured again to measure their specific growth rates (Supplementary Fig. S4). As a result, two strains showing the highest specific growth rates in the presence of 4 g L^−1^ Cys·HCl·H_2_O from each of ALE2 and ALE5, namely ALE2_1 (0.12 h^−1^) and ALE2_10 (0.15 h^−1^) from ALE2 and ALE5_2 (0.16 h^−1^) and ALE5_10 (0.11 h^−1^) from ALE5, were selected.

Further, ten colonies from the four selected strains grown on L agar plates were cultured again in the modified M9 medium containing 4 g L^−1^ Cys·HCl·H_2_O to measure their growth. In the case of the strains obtained from ALE2_10, four strains, ALE2_10_3, ALE2_10_6, ALE2_10_7, and ALE2_10_8, exhibited the highest specific growth rate in the presence of 4 g L^−1^ Cys·HCl·H_2_O; their specific growth rates were 0.13, 0.13, 0.13, and 0.14 h^−1^, respectively. Among them, the ALE2_10_3 strain showed the highest OD_660_ value at 12 h, which corresponds to the initial increase of cell growth after starting the cultivation, and was selected as a cysteine-resistant strain for further analysis (Supplementary Fig. S4a). Moreover, ALE5_2_9 and ALE5_10_4 were selected from the ALE5_2 and ALE5_10, respectively, as cysteine-resistant mutants for further analysis (Supplementary Fig. S4b), as they demonstrated specific growth rates of 0.15 and 0.16 h^−1^, respectively. No strains were selected for further analysis from ALE2_1, as no strains exhibited a higher specific growth rate higher than that of the original ALE2_1 (approximately 0.12 h^−1^) in the presence of 4 g L^−1^ Cys·HCl·H_2_O (Supplementary Fig. S4a).

**Fig. 2:**
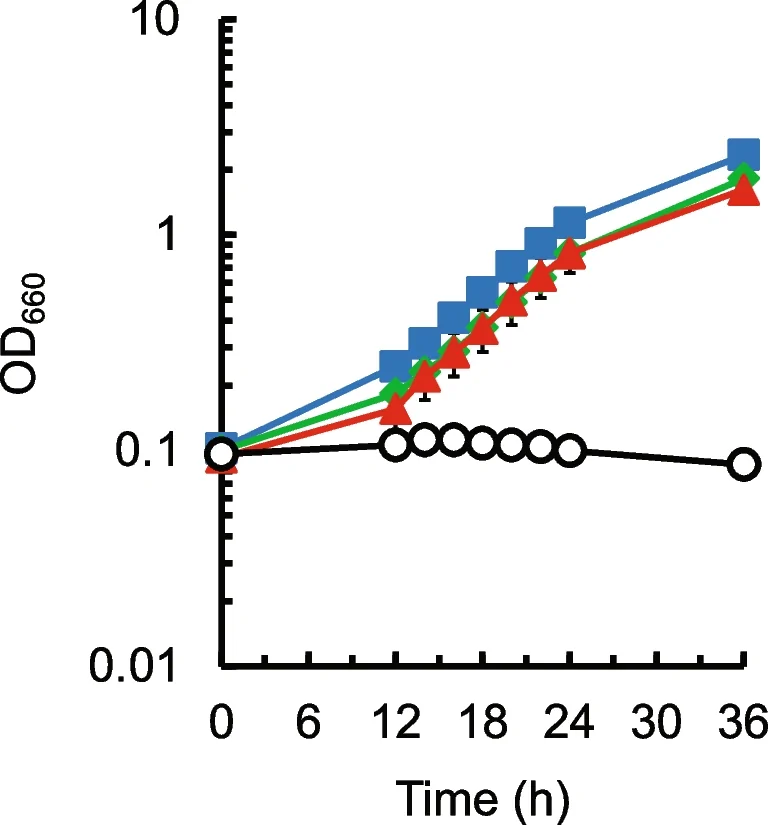
Cysteine resistance of the mutants obtained from the *aecD* gene disruptant of *C. glutamicum* via ALE. Cells cultured in the absence (the *aecD* gene disruptant) or presence (the isolated mutants) of 4 g L^−1^ Cys·HCl·H_2_O for 24 h were grown in a modified M9 medium containing 4 g L^−1^ Cys·HCl·H_2_O to confirm cysteine resistance of the isolated mutants. Cell growth of the *aecD* disruptant (black circles) and the mutants ALE2_10_3 (red triangles), ALE5_2_9 (blue squares), and ALE5_10_4 (green diamonds) is shown. The average ± standard deviation of the data in three independent cultures is shown

For confirmation of cysteine resistance of the isolated mutants, ALE2_10_3, ALE5_2_9, and ALE5_10_4 strains cultured in the presence of 4 g L^−1^ Cys·HCl·H_2_O were inoculated into the same medium and cultured for 24 h. As shown in Fig. 2, all the mutants grew well in the presence of 4 g L^−1^ Cys·HCl·H_2_O, and specific growth rates of the ALE2_10_3, ALE5_2_9, and ALE5_10_4 strains during exponential growth (12–24 h) were 0.14 ± 0.01, 0.13 ± 0.001, and 0.13 ± 0.002 h^−1^, respectively. From the results, the cysteine resistance of three strains was successfully confirmed.

### Identification of mutations related to cysteine resistance in *C. glutamicum*

To identify the mutations responsible for cysteine resistance in the cysteine-resistant mutants obtained by ALE, whole genome resequencing of the cell populations in ALE cultures and cells of the cysteine-resistant mutants was conducted. Here, the genomic DNA obtained from the cell populations from ALE2 and ALE5 at the end of ALE experiments (2279 h; 362 and 352 generations, respectively), the cysteine-resistant mutants of ALE2_10_3, ALE5_2_9, and ALE5_10_4, and the *aecD* disruptant was resequenced. In addition, the genome of the cell populations from ALE2 at 1655.8 h (248 generations) and ALE at 1680.5 h (240 generations) was also resequenced to compare the mutations in the cell populations just before increasing specific growth rates with those at the end of the ALE. The summary of the genome resequencing analyses is provided in Supplementary Table S2.

Genome resequencing analysis revealed mutations in the *ilvN* (NCgl1223) gene were detected in the genomes of both ALE2 and ALE5 cell populations and all the cysteine-resistant mutants isolated from the ALE cultures (Table 1). The *ilvN* gene encodes the regulatory small subunit of AHAS, which is involved in the biosynthesis of branched-chain amino acids (Keilhauer et al. 1993). The mutation found in the ALE2_10_3 strain, replacement of the 41st serine residue with proline (S41P) in the *ilvN* gene product, was also detected in cell populations from ALE2 at 248 and 362 generations. In contrast, the mutation found in the ALE5_2_9 and ALE5_10_4 strains, replacement of the 15th valine residue with isoleucine (V15I) in the *ilvN* gene product, was not detected in the cell population from ALE5 at 240 generations but was detected at 352 generations. The mutation, replacement of the 17th aspartic acid residue with glutamic acid (D17E) in the *ilvN* gene product, was found in both 240 and 352 generations of the ALE5 lineage. These results suggest that the mutations in the *ilvN* gene corresponding to S41P and D17E substitutions seem responsible for adaptation to the conditions where the cells grow in the presence of 1.5–3 g L^−1^ Cys·HCl·H_2_O.

**Table 1:** Mutations found in the *ilvN* gene of the ALE cell populations and isolated cysteine-resistant mutants

Cell population of ALE or cysteine-resistant strain	Mutation found in *ilvN* locus		
	Position on the genome	Mutation	Corresponding amino acid substitution
Cell population from ALE2 at 1655.8 h (248 generations)	1340145	T → C	Ser41 → Pro
Cell population from ALE2 at 2279 h (362 generations)	1340145	T → C	Ser41 → Pro
ALE2_10_3 strain	1340145	T → C	Ser41 → Pro
Cell population from ALE5 1680.5 h (240 generations)	1340075	C → G	Asp17 → Glu
Cell population from ALE5 at 2279 h (352 generations)	1340067	G → A	Val15 → Ile
	1340075	C → G	Asp17 → Glu
ALE5_2_9 strain	1340067	G → A	Val15 → Ile
ALE5_10_4 strain	1340067	G → A	Val15 → Ile

**Fig. 3:**
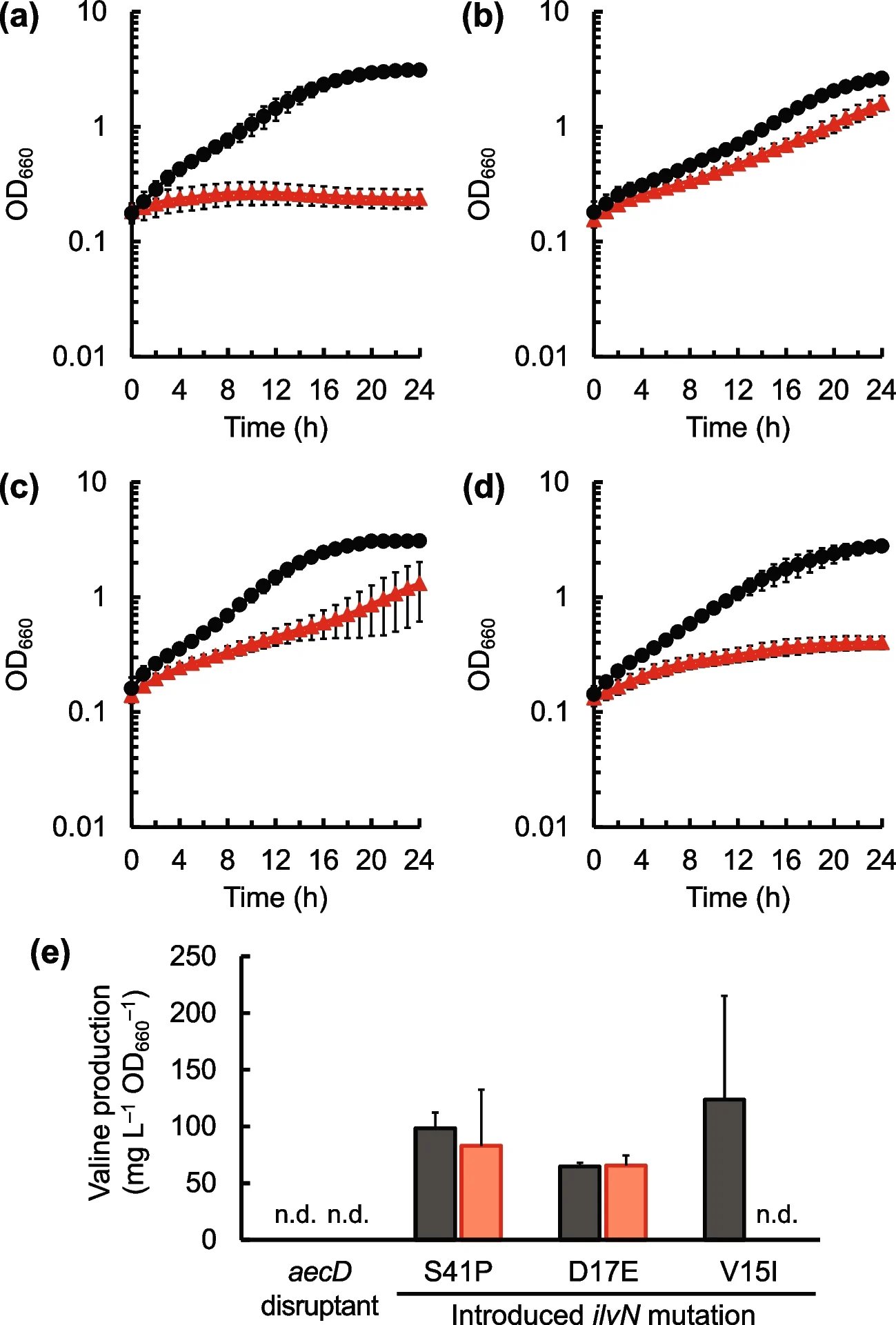
Effect of introduction of *ilvN* mutations into the *aecD* gene disruptant of *C. glutamicum* on growth and valine production in the presence of cysteine. Cell growth of the *aecD* disruptant (**a**) and the *aecD* disruptant carrying the mutations S41P (**b**), D17E (**c**), and V15I (**d**) in *ilvN* gene in modified M9 medium without cysteine addition (black circles) and with the addition of 0.5 g L^−1^ Cys·HCl·H_2_O (red triangles) is shown. In addition, valine concentration in culture supernatant obtained from the culture experiments at 24 h in (a)–(d) in the absence (black bars) and presence (red bars) is shown (**e**). The average ± standard deviation of the data in three independent cultures is shown

To clarify which mutation(s) in the *ilvN* gene were related to cysteine resistance in the cysteine-resistant mutants, each identified mutation was introduced into the *ilvN* locus in the genome of the *aecD* gene disruptant and the growth of the constructed strains in the presence of cysteine was evaluated. As shown in Fig. 3a–d, the introduction of the *ilvN* mutations corresponding to S41P and D17E resulted in improved cell growth in the presence of 0.5 g L^−1^ Cys·HCl·H_2_O, while the introduction of the other *ilvN* mutation corresponding to V15I improved the growth slightly. These results indicate that the *ilvN* mutations S41P and D17E are responsible for resistance to cysteine in *C. glutamicum*.

### Effect of branched-chain amino acids on growth in the presence of cysteine in *C. glutamicum*

The concentration of branched-chain amino acids in the culture supernatant obtained at 24 h, shown in Fig. 3a–d, was quantified because the *ilvN* gene product is involved in branched-chain amino acid biosynthesis. As shown in Fig. 3e, valine was detected in the culture supernatants of strains carrying the *ilvN* mutations S41P and D17E both with and without the addition of cysteine, whereas for the strain carrying the *ilvN* mutation V15I, valine was detected in the supernatant without cysteine addition, but this was not observed in the presence of cysteine. The other branched-chain amino acids, leucine and isoleucine, were not detected in the culture supernatants of any of the strains examined (data not shown). These results indicate that *ilvN* mutations are related to increased valine formation in *C. glutamicum*.

**Fig. 4:**
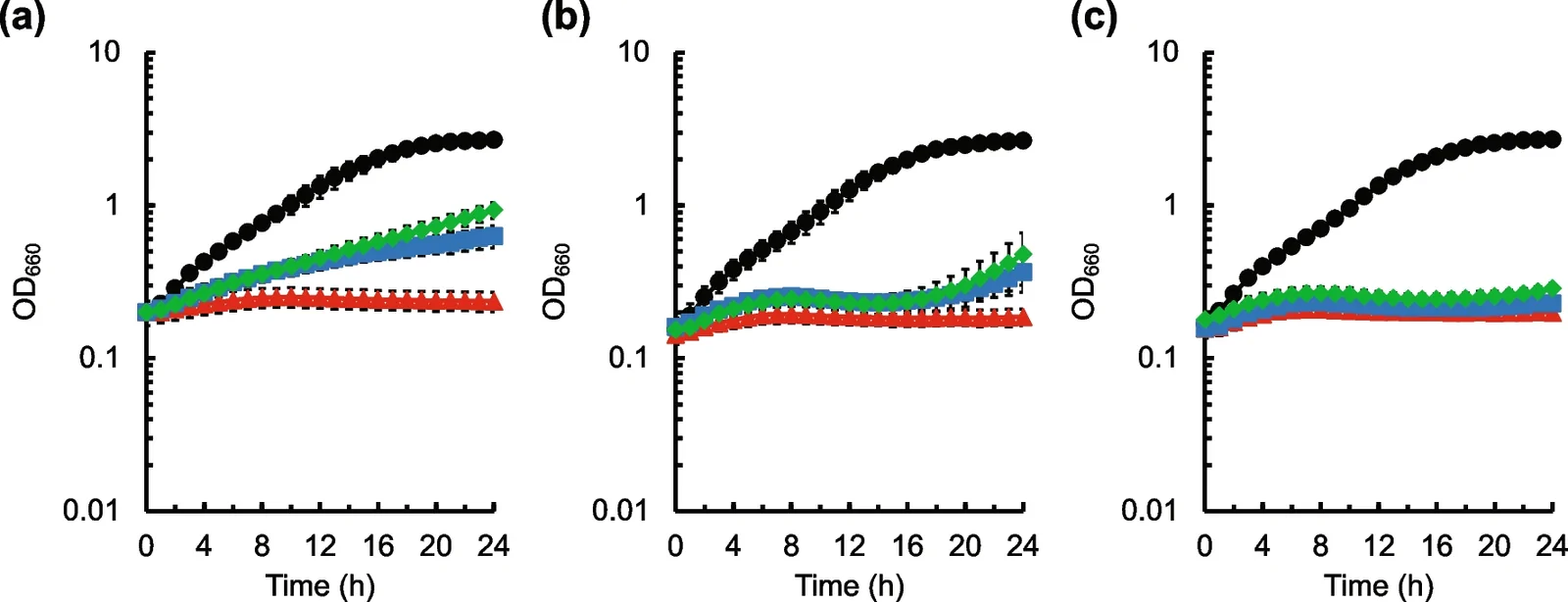
Effect of branched-chain amino acids on growth of the *aecD* gene disruptant of *C. glutamicum* in the presence of cysteine. Growth of the *aecD* gene disruptant of *C. glutamicum* in modified M9 medium without cysteine addition (black circles) and with addition of 0.5 g L^−1^ Cys·HCl·H_2_O alone (red triangles) and 0.5 g L^−1^ Cys·HCl·H_2_O plus 0.1 g L^−1^ (blue squares) or 0.2 g L^−1^ (green diamonds) valine (**a**), leucine (**b**), or isoleucine (**c**) is shown. The average ± standard deviation of the data in three independent cultures is shown

It can be speculated that the enhancement of valine formation by *ilvN* mutations mitigates growth inhibition by cysteine in *C. glutamicum*. Therefore, the effect of valine on the growth inhibitory effect of cysteine in *C. glutamicum* was investigated. As shown in Fig. 4, the addition of 0.1 g L^−1^ valine improved the cell growth of the *aecD* gene disruptant in the presence of 0.5 g L^−1^ Cys·HCl·H_2_O, and further improvement of growth was observed upon 0.2 g L^−1^ valine addition. The addition of leucine slightly improved the growth in the presence of Cys·HCl·H_2_O, while the addition of isoleucine did not. These results indicate that valine can mitigate the growth inhibitory effect of cysteine in *C. glutamicum*, and that leucine can slightly suppress it.

### Effect of *ilvN* mutations on amino acid production in *C. glutamicum*

To investigate the effect of *ilvN* mutations on the biosynthesis of amino acids other than branched-chain amino acids, amino acids in the culture supernatant of the *ilvN* mutation-carrying *aecD* gene disruptant were measured (Supplementary Fig. S5). Since the detection sensitivity of the measurement system for branched-chain amino acids described above was low, we used another precolumn amino acid measurement system with high detection sensitivity.

Using this measurement system, leucine could be detected in the culture supernatant of the *aecD* gene disruptant carrying the mutant *ilvN* genes, whereas it was not detected in the culture supernatant of the parental *aecD* gene disruptant. This result indicates that the *ilvN* mutations result in increased production of leucine as well as valine. However, its production levels were lower than those of valine production. Moreover, glycine production was detected as well in the *ilvN* mutation-carrying *aecD* gene disruptant whereas it was not detected in the *aecD* gene disruptant, suggesting that the *ilvN* mutations might affect glycine biosynthesis. In addition, histidine production by the *aecD* gene disruptant was detected, but it was not detected in the *ilvN* mutation-carrying *aecD* gene disruptant, suggesting that *ilvN* mutations might negatively affect histidine biosynthesis.

### The *katA* gene encoding catalase is not related to the mitigation of growth inhibitory effect by valine

As described in the “Introduction” section, cysteine is related to the induction of oxidative stress by the Fenton reaction because it can reduce Fe^3+^ to Fe^2+^. In the Fenton reaction, hydroxyl radical is produced from hydrogen peroxide using Fe^2+^ as an oxidant. If valine induces the synthesis of catalase, which catalyzes the degradation of hydrogen peroxide to water and oxygen, and synthesized catalase enhances the degradation of hydrogen peroxide, the chance to occur the Fenton reaction with Fe^2+^ produced by the reduction of Fe^3+^ with cysteine would be reduced, and as a result, growth inhibitory effect of cysteine would be mitigated. Therefore, we investigated the effects of valine addition and *ilvN* mutations on *katA* gene expression in *C. glutamicum *with RT-qPCR.

As shown in Supplementary Fig. S6, the *katA* gene expression was not significantly changed by the addition of 0.1 g L^−1^ valine in the *aecD* disruptant of *C. glutamicum*. Moreover, the *katA* gene expression in the *aecD* disruptant carrying *ilvN* S41P mutation was lower than that in the parental *aecD* disruptant, whereas the *katA* gene expression in the *aecD* disruptant carrying *ilvN* D17E and V15I mutations was not significantly different from that in the parental *aecD* disruptant (Supplementary Fig. S6). These results indicate that catalase does not seem related to the mitigation of growth inhibition by cysteine in *C. glutamicum*.

Moreover, the effect of *katA* gene disruption on the mitigation of the growth inhibitory effect of cysteine by valine was investigated. As shown in Supplementary Fig. S7, valine could improve the decreased growth in the presence of 0.5 g L^−1^ Cys·HCl·H_2_O even though the *katA* gene was disrupted in the *aecD* disruptant of *C. glutamicum*. This result indicates that the mitigation of the growth inhibitory effect of cysteine by valine is not related to oxidative stress in *C. glutamicum*.

### Effect of cysteine on activity of AHAS from *C. glutamicum*

It could be speculated that growth inhibition cysteine was caused by inhibition of AHAS activity which resulted in perturbation to valine biosynthesis. Therefore, the activity of AHAS from the *aecD* gene disruptant in the presence of cysteine was measured. As shown in Supplementary Fig. S8, the activity of AHAS from the *aecD* disruptant was reduced by about 40% with 0.1 g L^−1^ Cys·HCl·H_2_O, indicating that cysteine inhibits AHAS activity in *C. glutamicum*. Considering this result, the growth inhibitory effect of cysteine in *C. glutamicum* is related to the inhibition of AHAS activity by cysteine. As expected, the AHAS activity was inhibited by 100 mM valine.

Moreover, the activity of AHAS from the *aecD* disruptant harboring *ilvN* mutations in the presence of valine and cysteine was also investigated. As shown in Supplementary Fig. S8, the activity of AHAS from the *aecD* disruptant carrying *ilvN* mutations (i.e., S41P, D17E, and V15I) was not inhibited by valine, indicating that this phenomenon is consistent with enhanced valine production in the *aecD* gene disruptant carrying *ilvN* mutations. Interestingly, the activity of AHAS from the *aecD* disruptant carrying the *ilvN* S41P mutation was inhibited by 0.1 g L^−1^ Cys·HCl·H_2_O, but more than 80% of the activity remained. Similarly, 0.1 g L^−1^ Cys·HCl·H_2_O inhibited the activity of AHAS from the *aecD* disruptant carrying the *ilvN* mutations D17E and V15I, but around 70% of the activity remained. These results indicate that AHAS containing the mutant IlvN protein as a small subunit exhibits resistance to cysteine, and this phenomenon is caused by cysteine resistance of the *ilvN* mutation-carrying *aecD* gene disruptant of *C. glutamicum*.

### Cysteine production by engineered cysteine-resistant mutants

Finally, the effectiveness of cysteine-resistant mutants obtained by ALE as bioproduction hosts was investigated. In the present study, cysteine production by engineered cysteine-resistant mutants was examined. The plasmid pCYS*_Cg(T94A)-SER* carrying the mutant *cysE* and *serA* genes from *C. glutamicum* (Kondoh and Hirasawa 2019) was introduced into the cysteine-resistant mutants ALE2_10_3, ALE5_2_9, and ALE_5_10_4 as well as the parental *aecD* gene disruptant, and cysteine production by the constructed recombinant strains was evaluated. In the experiments, yeast extract was added to the production medium because the addition of yeast extract enhances cell growth and cysteine production as reported previously (Kondoh and Hirasawa 2019).

**Fig. 5:**
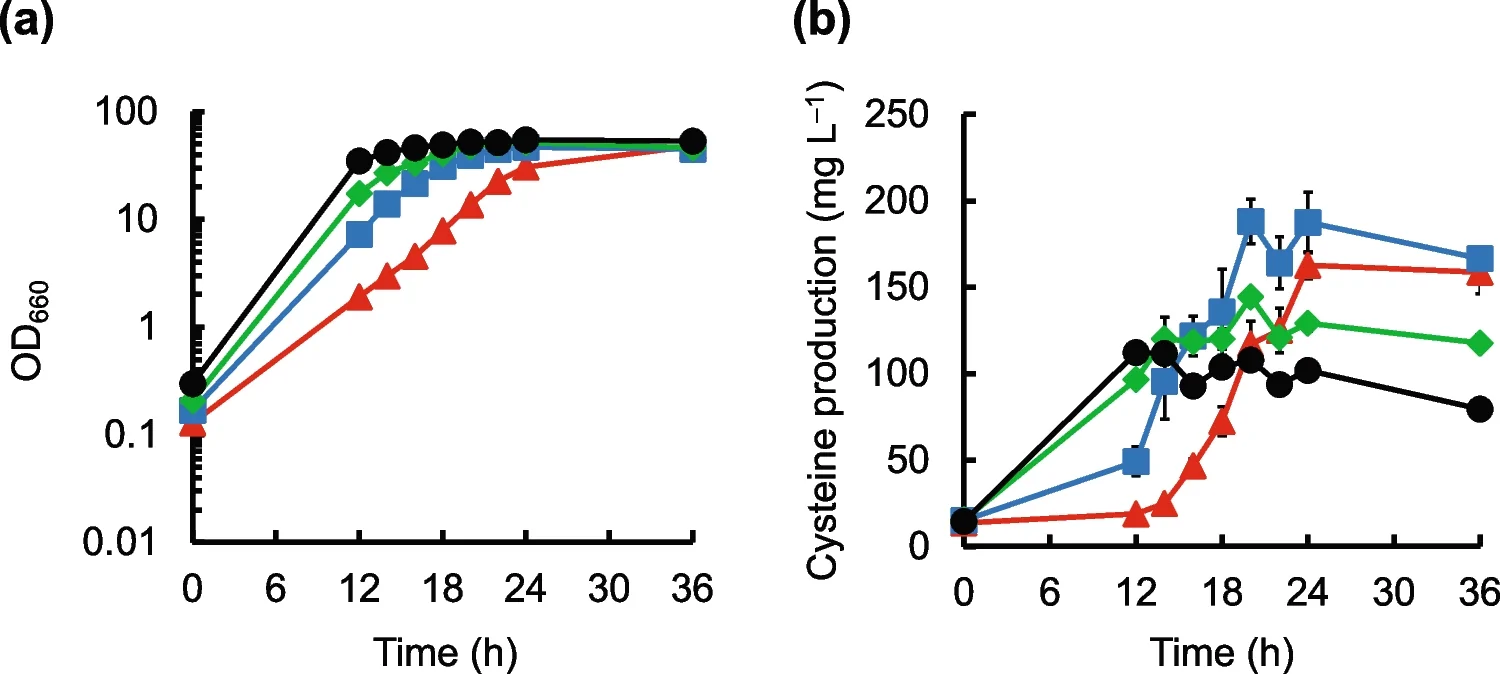
Cysteine production by the engineered cysteine-resistant mutants of *C. glutamicum*. Time courses of cell growth (**a**) and cysteine production (**b**) in the *aecD* disruptant (black circles), ALE2_10_3 (red triangles), ALE5_2_9 (blue squares), and ALE5_10_4 (green diamonds) transformed with pCYS*_Cg(T94A)-SER* are shown. For cultivation, a semisynthetic cysteine production medium was used. The average ± standard deviation of the data in three independent cultures is shown

Cell growth of the cysteine-resistant mutants ALE2_10_3, ALE5_2_9, and ALE_5_10_4 transformed with pCYS*_Cg(T94A)-SER* was lower than that of the *aecD* gene disruptant transformed with the same plasmid (Fig. 5a). The cysteine production levels in the pCYS*_Cg(T94A)-SER*-transformed cysteine-resistant mutants were higher than those in the pCYS*_Cg(T94A)-SER*-transformed *aecD* gene disruptant (Fig. 5b). This indicates that cysteine resistance is an effective phenotype for cysteine production by *C. glutamicum*.

## Discussion

In this study, the mechanism for growth inhibition by cysteine in *C. glutamicum* was investigated by analyzing the cysteine-resistant mutants isolated via ALE in which Cys·HCl·H_2_O concentration was increased from 1.5 to 4 g L^−1^ stepwise. From this study, it was found that the mutations in the *ilvN* gene encoding the regulatory small subunit of AHAS were a substantial factor contributing to cysteine resistance in the cysteine-resistant mutants of *C. glutamicum.* Furthermore, it was found that these mutations were associated with enhanced valine biosynthesis. The *ilvN* mutations were found in the genome of the cell populations both before and after increasing specific growth rate in the presence of 4 g L^−1^ Cys·HCl·H_2_O and the isolated cysteine-resistant mutants (Table 1), indicating that the *ilvN* mutations are related to initial adaptation to cysteine in ALE in the presence of less than 4 g L^−1^ Cys·HCl·H_2_O.

*C. glutamicum* has one AHAS (Keilhauer et al. 1993), whereas *E. coli* has three (Wek et al. 1985; Lawther et al. 1987; Gollop et al. 1989). Among the three AHASs from *E. coli*, AHAS III shows high homology with the AHAS from *C. glutamicum*, and the regulatory subunit of AHAS III, which is encoded by *ilvH* gene, has an N-terminal ACT domain (Chipman and Shaanan 2001). Branched-chain amino acids bind to the regulatory subunit of AHAS and inhibit AHAS activity (Gedi and Yoon 2012). The crystal structure of the regulatory subunit of AHAS III from *E. coli* revealed that it becomes a dimer, and the 11th and 29th asparagine residues in individual monomers and the 9th leucine, 16th leucine, and 35th valine residues in the ACT domain are related to valine binding (Kaplun et al. 2006). Multiple alignments of the amino acid sequences of the regulatory subunit of AHAS from various bacteria (Supplementary Fig. S9) revealed that the 17th aspartic acid and 41st serine residues in IlvN from *C. glutamicum* corresponded to the 11th asparagine and 35th valine residues in IlvH from *E. coli*. Indeed, D17E and S41P mutations in IlvN found in the cysteine-resistant mutants of *C. glutamicum* resulted in feedback resistance of AHAS with valine and increased in valine production without addition of cysteine. Guo et al. (2015) showed that the substitution of the 41st serine residue in IlvN with valine resulted in feedback resistance of AHAS to valine and increased production of branched-chain amino acids in *C. glutamicum*. Furthermore, we found that cysteine inhibited AHAS activity in *C. glutamicum* and that the AHAS containing the mutant IlvN S41P or D17E as a small subunit exhibited resistance to cysteine (Supplementary Fig. S8). Considering the results obtained in this study, cysteine reduces valine biosynthesis because of the decreased activity of AHAS, which results in growth inhibition by cysteine in *C. glutamicum*.

As for the V15I mutation in IlvN, multiple alignment analysis revealed that the amino acid residues in the homologs from other bacteria corresponding to the 15th valine residue in IlvN from *C. glutamicum* are branched-chain amino acids. This mutation also led to resistance of AHAS to not only valine but also cysteine (Supplementary Fig. S8). The *aecD* disruptant carrying *ilvN* V15I mutation produced valine in the absence of cysteine, but valine production was not observed in the strain carrying this mutation in the presence of cysteine (Fig. 3d). The results indicate that the V15I mutation in the IlvN result in resistance of AHAS against valine and cysteine, but does not result in valine production in the presence of cysteine.

Additionally, in the *ilvN* mutation-carrying *aecD* gene disruptant, production of leucine and glycine was observed using an amino acid analysis system with high detection sensitivity, but they were low compared with valine production (Supplementary Fig. S5). Leucine production is consistent with the function of IlvN in AHAS, but the mechanism of glycine production is obscure. Increased branched-chain amino acid production caused by *ilvN* mutations might affect glycine biosynthesis. Further researches on the relationship between branched-chain amino acid biosynthesis and glycine biosynthesis will be required.

In addition to the *ilvN* mutations, other mutations were found in the *zupT* (NCgl1379) gene locus within the genomes of cell populations from ALE2 and ALE5 before and after increasing the specific growth rate in the presence of 4 g L^−1^ Cys·HCl·H_2_O (Supplementary Table S3). The mutations in the *zupT* gene were also detected in the cysteine-resistant mutants (Supplementary Table S3). The ZupT homolog from *E. coli* is a member of the Zrt- and Irt-like protein (ZIP) transporter family and is involved in the uptake of zinc and other metals, such as iron and cadmium (Grass et al. 2002, 2005). ZupT from *E. coli* contains eight transmembrane domains (TM1–TM8) and two metal-binding sites (M1 and M2). Both metal-binding sites have ligands from TM4 and TM5, and M2 has a ligand (a glutamic acid residue) from TM6, suggesting that TM4–TM6 are responsible for the metal transport activity of ZupT (Roberts et al. 2021). Multiple alignment analysis of the ZIP family protein homologs shows that TM1–TM8 in ZupT from *C. glutamicum* and the amino acid residues constituting M1 and M2 are conserved (Supplementary Fig. S10). In the cell population and cysteine-resistant mutants of *C. glutamicum* obtained via ALE in the present study, mutations were found in the 8th phenylalanine, 19th alanine, and 267th phenylalanine residues, all of which are not conserved in the ZupT homologs and are not located in TM4–TM6. Therefore, these mutations may not affect the activity of ZupT and may not be related to cysteine resistance in *C. glutamicum*. However, if the mutations in ZupT found in the cell population and the cysteine-resistant mutants reduce metal transport activity, the uptake of iron may be reduced, and as a result, the oxidative stress caused by the Fenton reaction in the presence of cysteine may be mitigated. Further investigations on the contribution of ZupT mutations to cysteine resistance in *C. glutamicum* are required.

Mutations that were not present in the cell population of ALE2 at 1655.8 h but present in the cell population of ALE2 at 2279 h and the isolated cysteine-resistant mutant ALE2_10_3 are shown in Supplementary Table S4. These mutations may be responsible for the resistance to high cysteine concentration (i.e., 4 g L^−1^ Cys·HCl·H_2_O). Among the genes carrying the mutation, *glmU* (NCgl0906) and NCgl2203 encode enzymes related to the biosynthesis of peptidoglycan components. The result suggests a relationship between cysteine resistance and peptidoglycan biosynthesis.

The number of genes that did not carry mutations in cell population of ALE5 at 1680.5 h (i.e., before increasing specific growth rate in the presence of 4 g L^−1^ Cys·HCl·H_2_O) but carry mutations in cell population of ALE5 at 2279 h (i.e., after increasing specific growth rate in the presence of 4 g L^−1^ Cys·HCl·H_2_O) and the isolated cysteine-resistant mutant ALE5_2_9 (Supplementary Table S5) was larger than that in the case of ALE series ALE2 (Supplementary Table S4). Among such genes, those related to DNA synthesis and repair were included (NCgl0641, NCgl1120, and *hrpA* (NCgl1852)) (Supplementary Table S5). These mutations may be responsible for the increased mutation rate, and as a result, the number of genes carrying mutations increased in the ALE5 series. Indeed, the number of mutations found in the cell population in ALE5 at 2279 h was greater than that in the cell population in ALE2 at 2279 h (Supplementary Table S2).

Cysteine resistance is expected to be a useful phenotype for cysteine production by microbial cells. We found that the utilization of cysteine-resistant mutants as hosts increased cysteine production compared to their parental strain. To enhance the production of useful materials using cysteine as a starting compound, the addition of cysteine to the culture medium will be effective. Therefore, it is expected that cysteine-resistant mutants will be effective hosts for producing such materials because a high concentration of cysteine can be added to the culture medium. Further studies on the production of useful materials from cysteine, such as glutathione and ergothioneine, will be required.

In conclusion, growth inhibition by cysteine is caused by inhibition of the biosynthesis of branched-chain amino acids, particularly valine biosynthesis, in *C. glutamicum*. However, other factors as well as branched-chain amino acid biosynthesis may contribute growth inhibitory effect of cysteine in *C. glutamicum*. Further investigations will be required to understand growth inhibitory effect of cysteine in microorganisms.

## Supplementary Information

Below is the link to the electronic supplementary material.

Supplementary file1 (PDF 5186 KB)

## Data Availability

All data generated or analyzed during this study except for raw data of genome resequencing are included in this published article and its supplementary information file. The fastq files obtained from genome resequencing analysis are available under accession numbers DRR613113, DRR613114, DRR613115, DRR613116, DRR613117, DRR613118, DRR613119, and DRR613120.
