# Supplementary material for: Mutations in the ilvN gene mitigate growth inhibitory effect of cysteine in Corynebacterium glutamicum
Source: Appl Microbiol Biotechnol. 2025 Mar 10;109:61. doi: 10.1007/s00253-025-13444-y (PMC11893703; doi:10.1007/s00253-025-13444-y)
Supplement: Supplementary file 1 [file 253_2025_13444_MOESM1_ESM.pdf]

Supplementary materials

Mutations in the *ilvN* gene mitigate growth inhibitory effect of cysteine in *Corynebacterium glutamicum*

Kazuho Matsuhisa, Katsuhiro Ogawa, Kento Komata, Takashi Hirasawa*

School of Life Science and Technology, Institute of Science Tokyo, 4259 Nagatsuta-cho, Midori-ku, Yokohama, Kanagawa 226-8501, Japan

*Corresponding author

thirasawa@life.isct.ac.jp

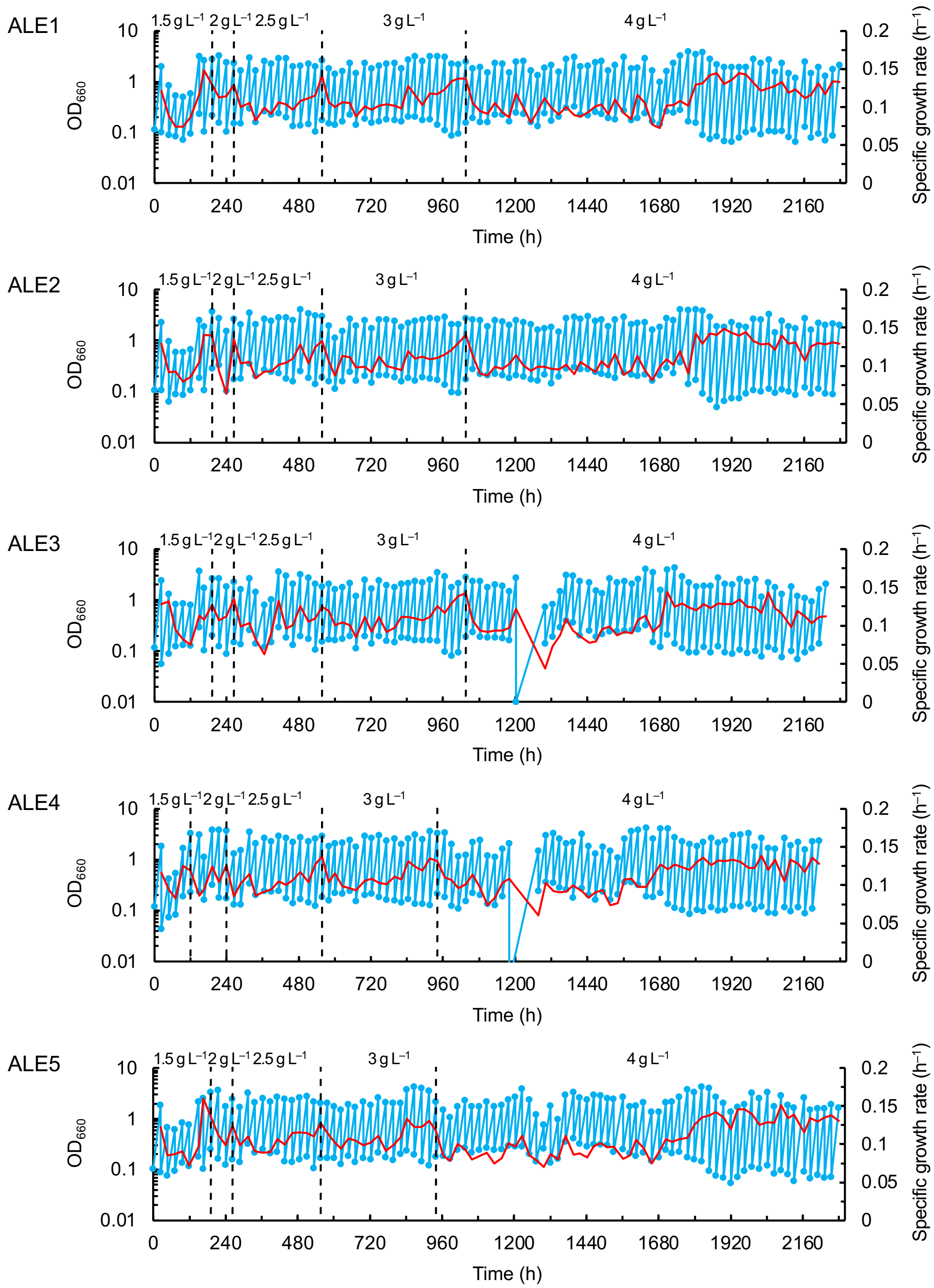

Supplementary Fig. S1 Adaptive laboratory evolution (ALE) of *C. glutamicum* in the presence of cysteine. Serial transfer of culture to the fresh medium was repeated and the concentration of cysteine hydrochloride monohydrate (Cys·HCl·H_2O) was increased stepwise. Blue circles with solid line and red line represent time courses of cell growth and specific growth rate, respectively. In addition, black dotted lines represent timing of changing the concentration of Cys·HCl·H_2O indicated above each graph.

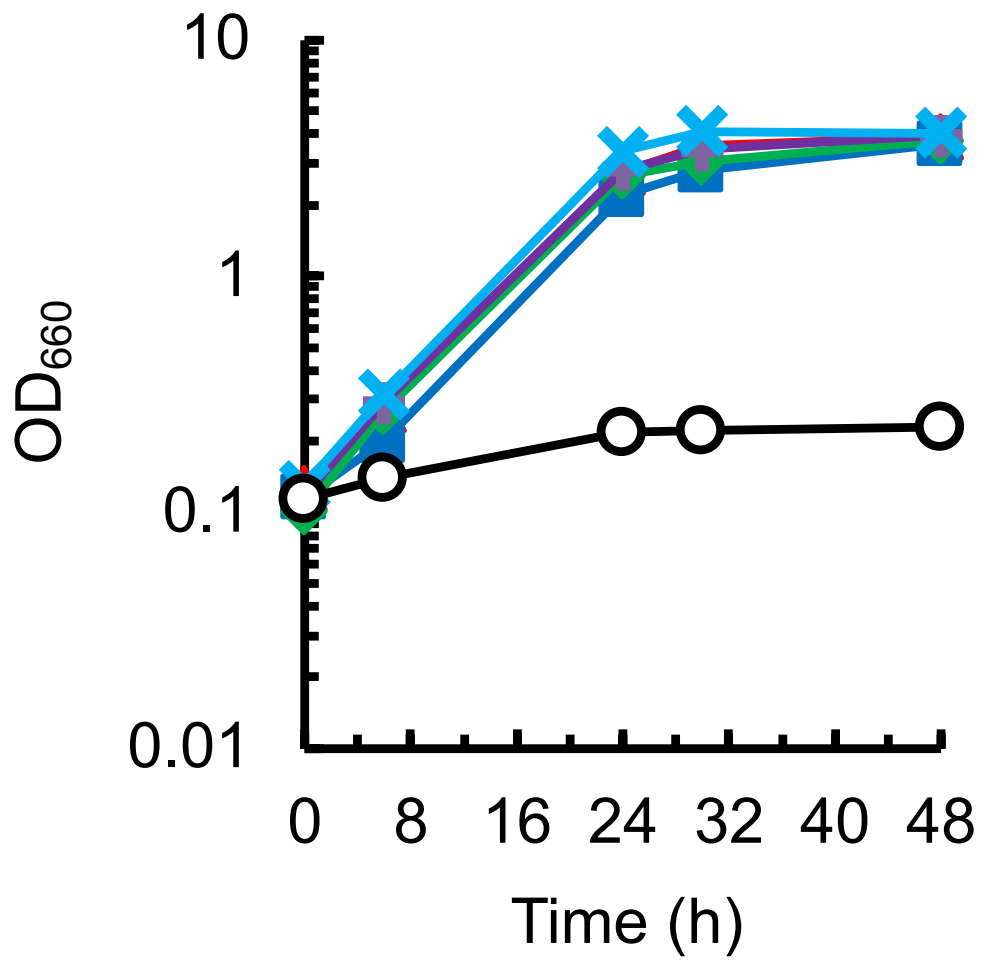

Supplementary Fig. S2 Growth of cell population obtained at the end of ALE in the presence of cysteine. Frozen cultures prepared at the end of ALE were inoculated to the modified M9 medium containing 4 g L^{-1} Cys·HCl·H_2O and cultured at 30°C for 1 d. For preparing the preculture of the *aecD* disruptant, the modified M9 medium without cysteine addition was used. The preculture was diluted in the modified M9 medium containing the same concentration of Cys·HCl·H_2O and then cultured at 30°C. Growth of the cells from ALE1 (red triangles), ALE2 (blue squares), ALE3 (green diamonds), ALE4 (purple pluses) and ALE5 (light blue crosses) as well as that of the *aecD* disruptant (black open circles) is shown.

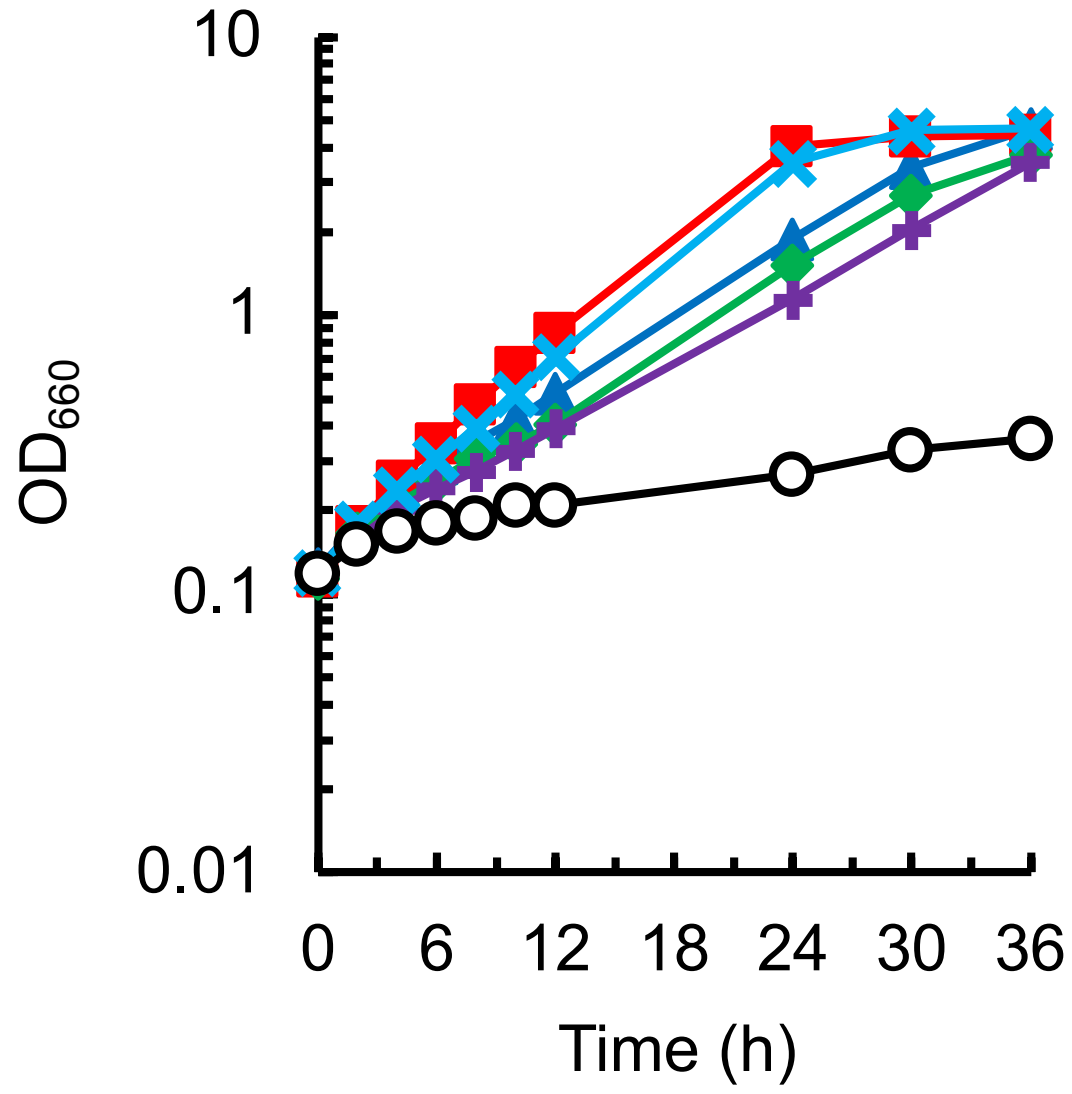

Supplementary Fig. S3 Stability of cysteine resistance of cell populations obtained by ALE. Frozen cultures prepared at the end of ALE were inoculated to the modified M9 medium and cultured at 30°C for 1 d. The preculture was diluted in the modified M9 medium and then cultured at 30°C. This serial transfer process was repeated five times. After that, the culture was diluted in the modified M9 medium containing 4 g L^{-1} Cys·HCl·H_2O and cultured at 30°C. Growth of the cells from ALE1 (red triangles), ALE2 (blue squares), ALE3 (green diamonds), ALE4 (purple pluses) and ALE5 (light blue crosses) as well as that of the *aecD* disruptant (black open circles) is shown.

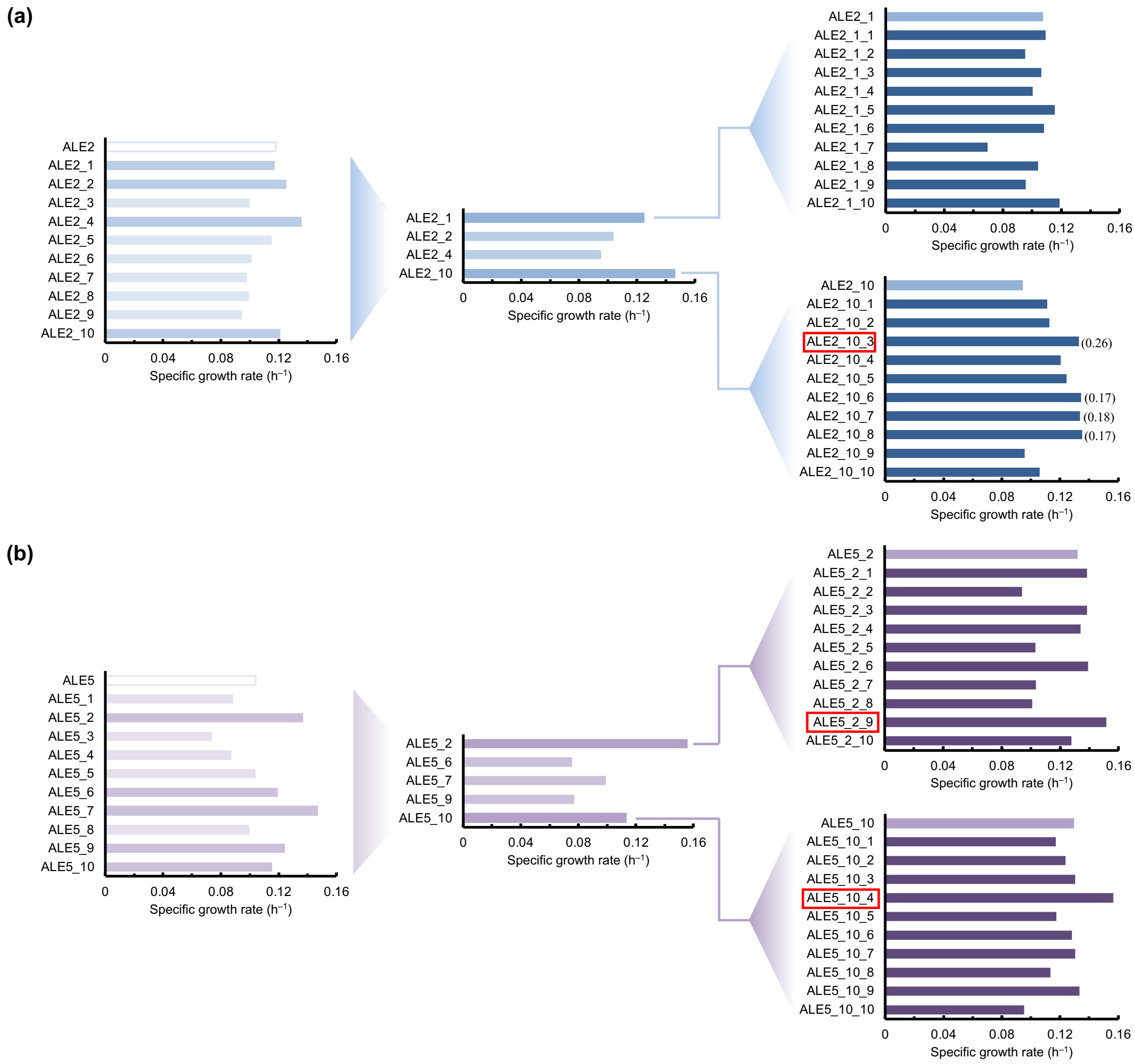

Supplementary Fig. S4 Isolation of cysteine-resistant mutant strains from cell populations at the end of ALE in ALE2 (a) and ALE5 (b). Values with parentheses for ALE2_10_3, ALE2_10_6, ALE2_10_7 and ALE2_10_8 represent the OD_{660} at 12 h. Red squares represent the strains chosen as cysteine-resistant mutants.

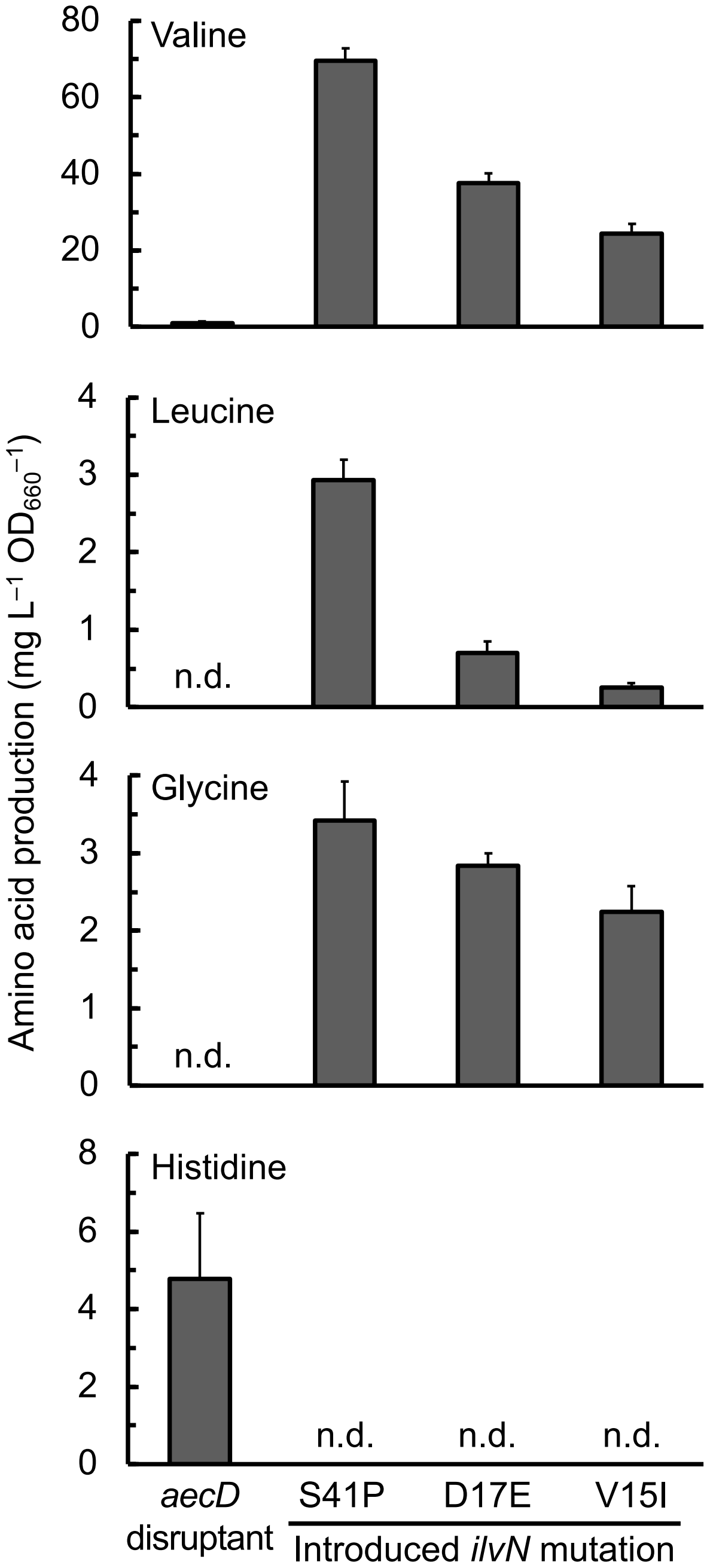

Supplementary Fig. S5 Effect of *ilvN* mutations on amino acid production in the *aecD* gene disruptant of *C. glutamicum*. The *aecD* gene disruptant and the ilvN mutation-carrying *aecD* gene disruptant were cultured in 4 mL of modified M9 medium for 24 h and then their culture supernatant was obtained. Amino acid concentration in the culture supernatant was determined using the precolumn amino acid analysis system. Average ± standard deviation in three independent cultures is shown. n.d., not detected.

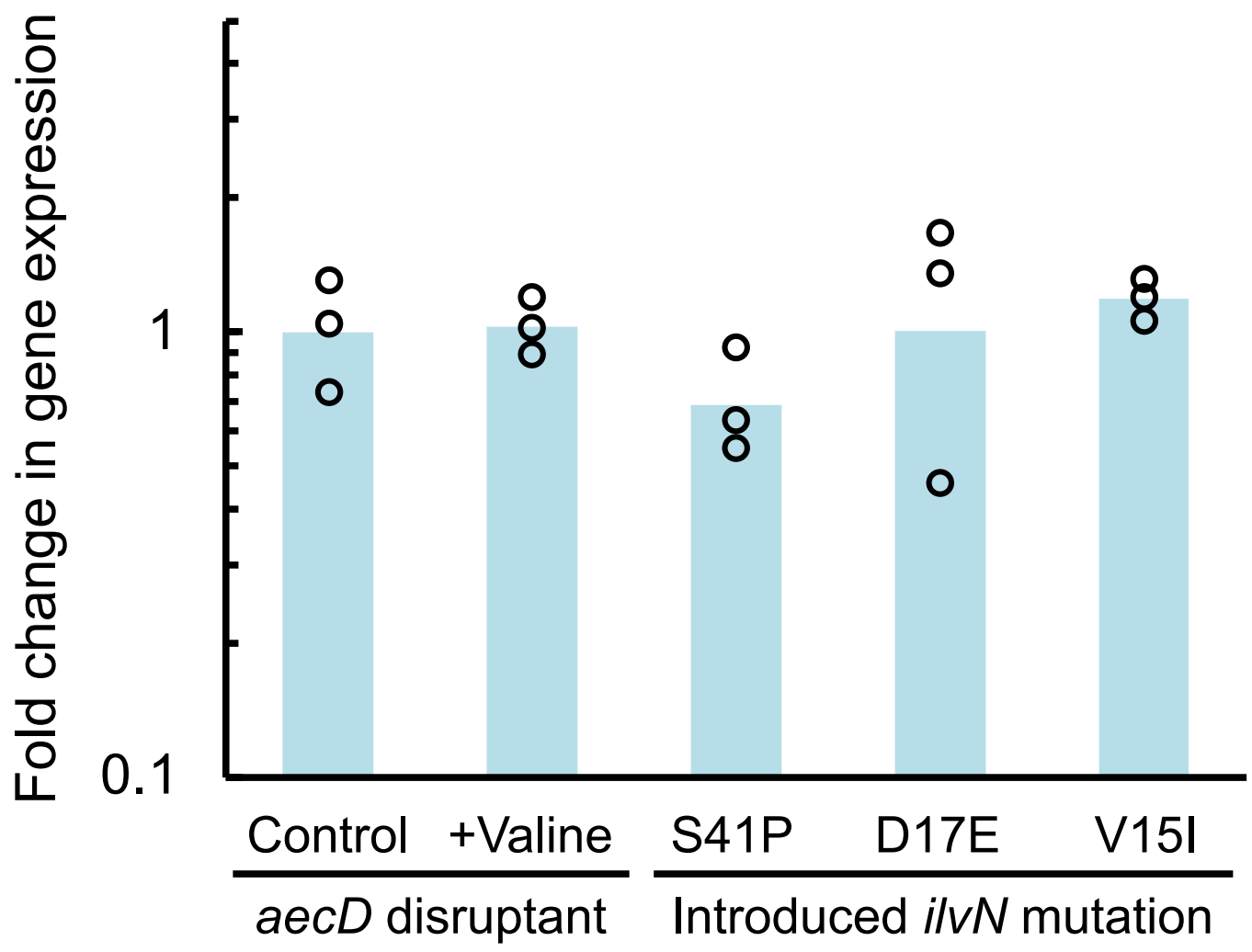

Supplementary Fig. S6 Effect of valine and *ilvN* mutations on *katA* gene expression in *C. glutamicum*. Geometric mean of fold change in *katA* gene expression by valine addition and introduction of *ilvN* mutation into the *aecD* gene disruptant relative to that in the *aecD* gene disruptant in three independent cultures is shown as bars. Circles represent the fold change in expression in each cell sample from three independent cultures of each strain.

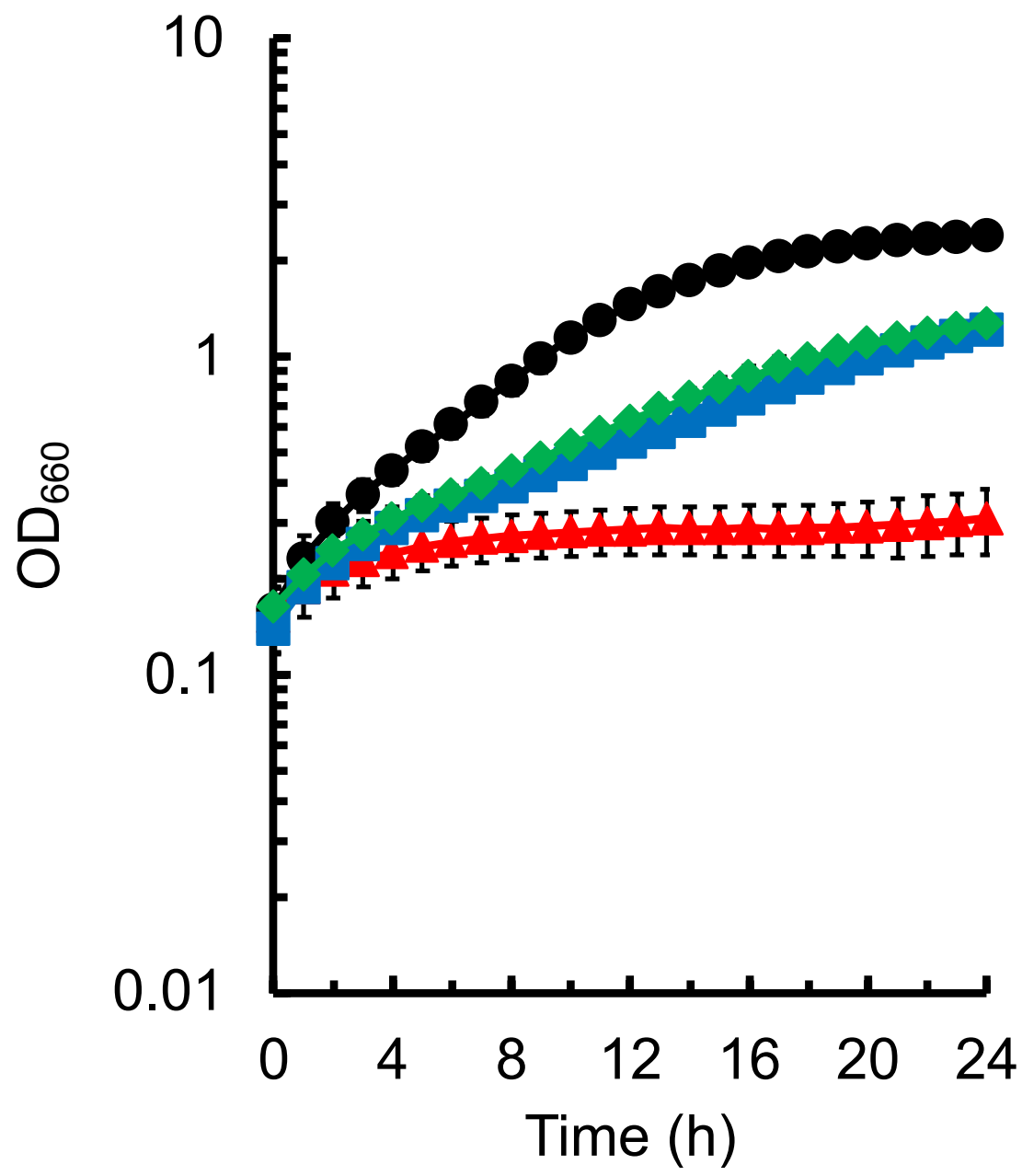

Supplementary Fig. S7 Effect of *katA* gene disruption on mitigation of growth inhibitory effect of cysteine by valine in *C. glutamicum*. Growth of the *aecD katA* disruptant of *C. glutamicum* without cysteine addition (black circles) and with addition of 0.5 g L^{-1} Cys·HCl·H_2O alone (red triangles) and 0.5 g L^{-1} Cys·HCl·H_2O plus 0.1 g L^{-1} (blue squares) or 0.2 g L^{-1} (green diamonds) valine is shown. Average ± standard deviation of the data in three independent culture is shown.

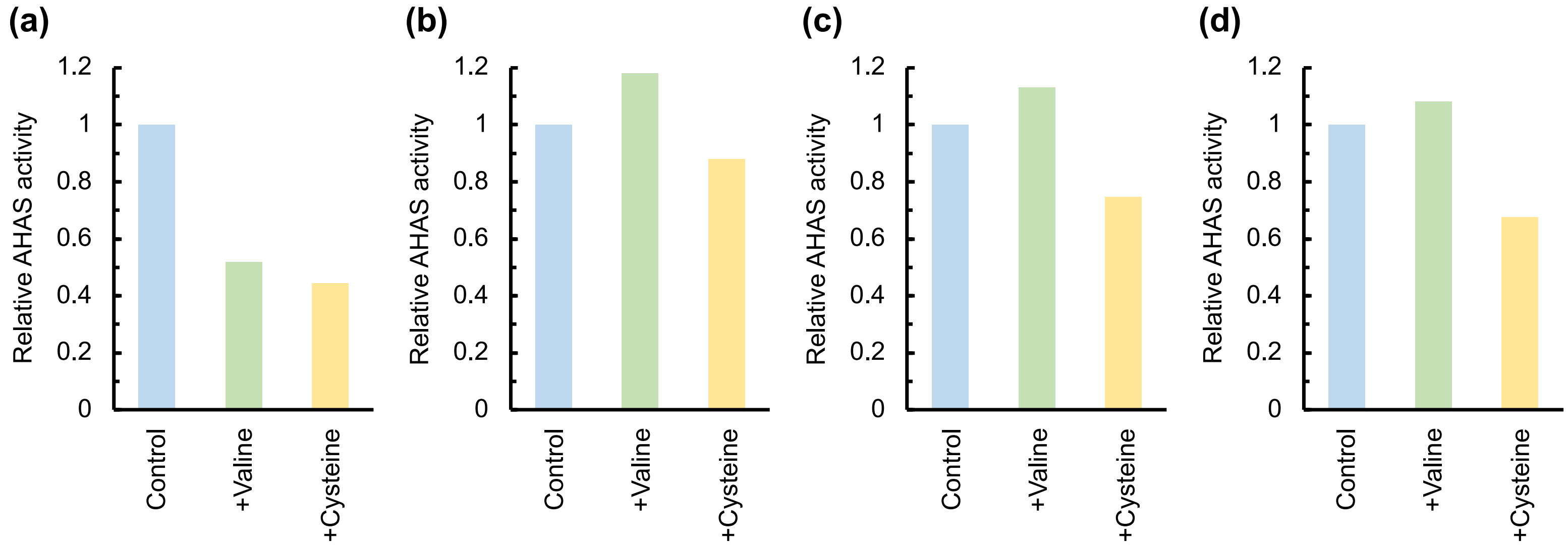

Supplementary Fig. S8 Effect of *ilvN* mutations on AHAS activity in *C. glutamicum*.
Activity of AHAS from the *aecD* gene disruptant (a) and *ilvN* mutations S41P (b), D17E (b) and V15I-carrying *aecD* gene disruptant in the presence of 1 mM valine (+Valine) and 0.1 g L^{-1} Cys·HCl·H_2O (+Cysteine) relative to that in the absence of them (Control) is shown.

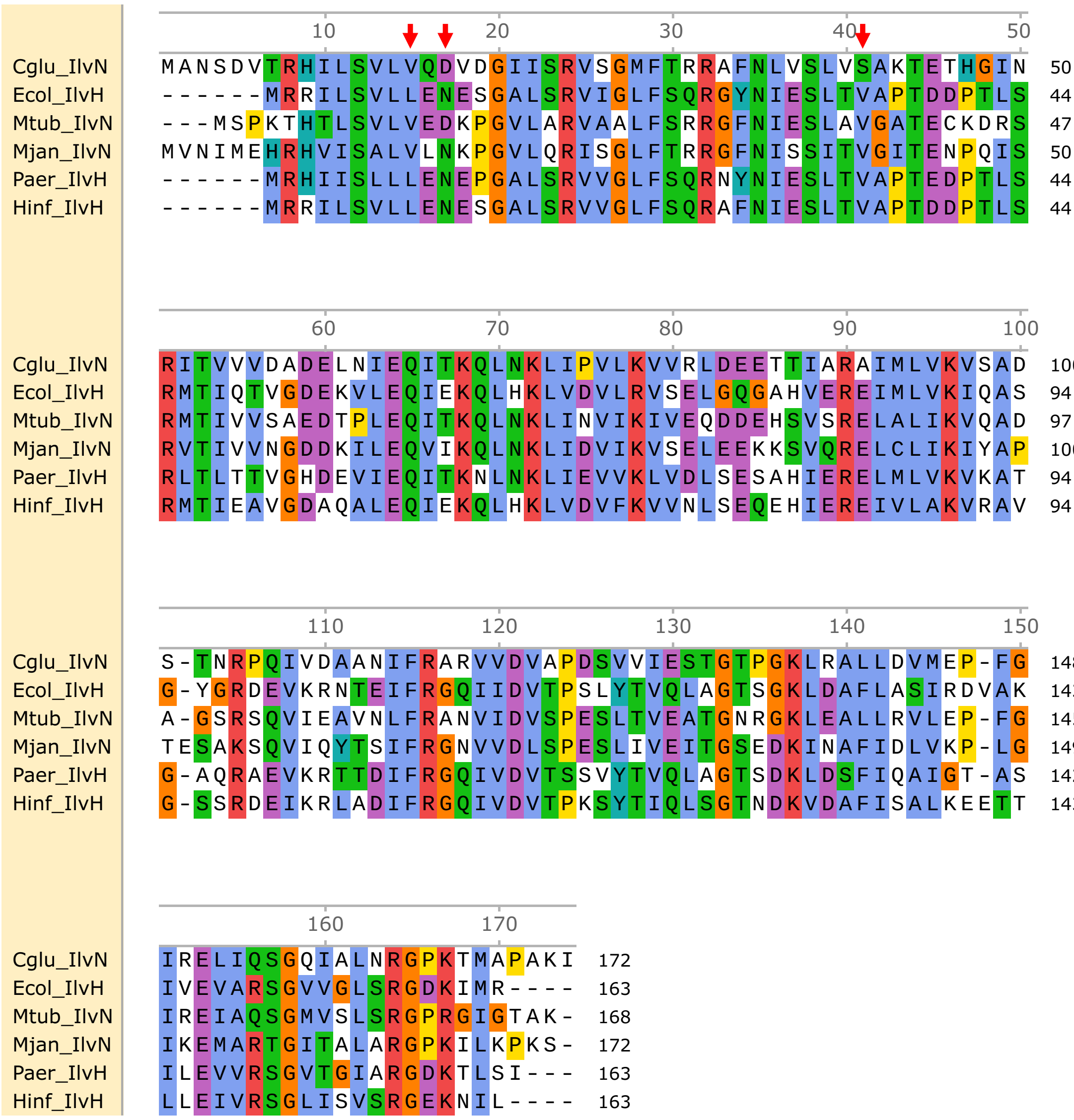

Supplementary Fig. S9 Multiple alignment of amino acid sequences of acetohydroxyacid synthase regulatory small subunits from *C. glutamicum* (Cglu_IlvN), *Escherchia coli* (Ecol_IlvN), *Mycobacterium tuberculosis* (Mtub_IlvN), *Methanocaldococcus jannaschii* (Mjan_IlvN), *Pseudomonas aeruginosa* (Paer_IlvH) and *Haemophilus influenzae* (Hinf_IlvN). Alignment analysis was performed using CLUSTAL omega (https://www.ebi.ac.uk/jdispatcher/msa/clustalo). The analysis data was visualized with SnapGene Viewer (GSL Biotech LLC, San Diego, CA). Coloring of amino acid residues is defined following the default Clustal X coloring scheme (https://support.snapgene.com/hc/en-us/articles/10527861802516-What-are-the-SnapGene-Protein-Multiple-Alignment-Color-Highlighting-Schemes). Red arrows represent the amino acid residues where the mutations were found in the cell population in ALE cultures and the cysteine-resistant mutants of *C. glutamicum*.

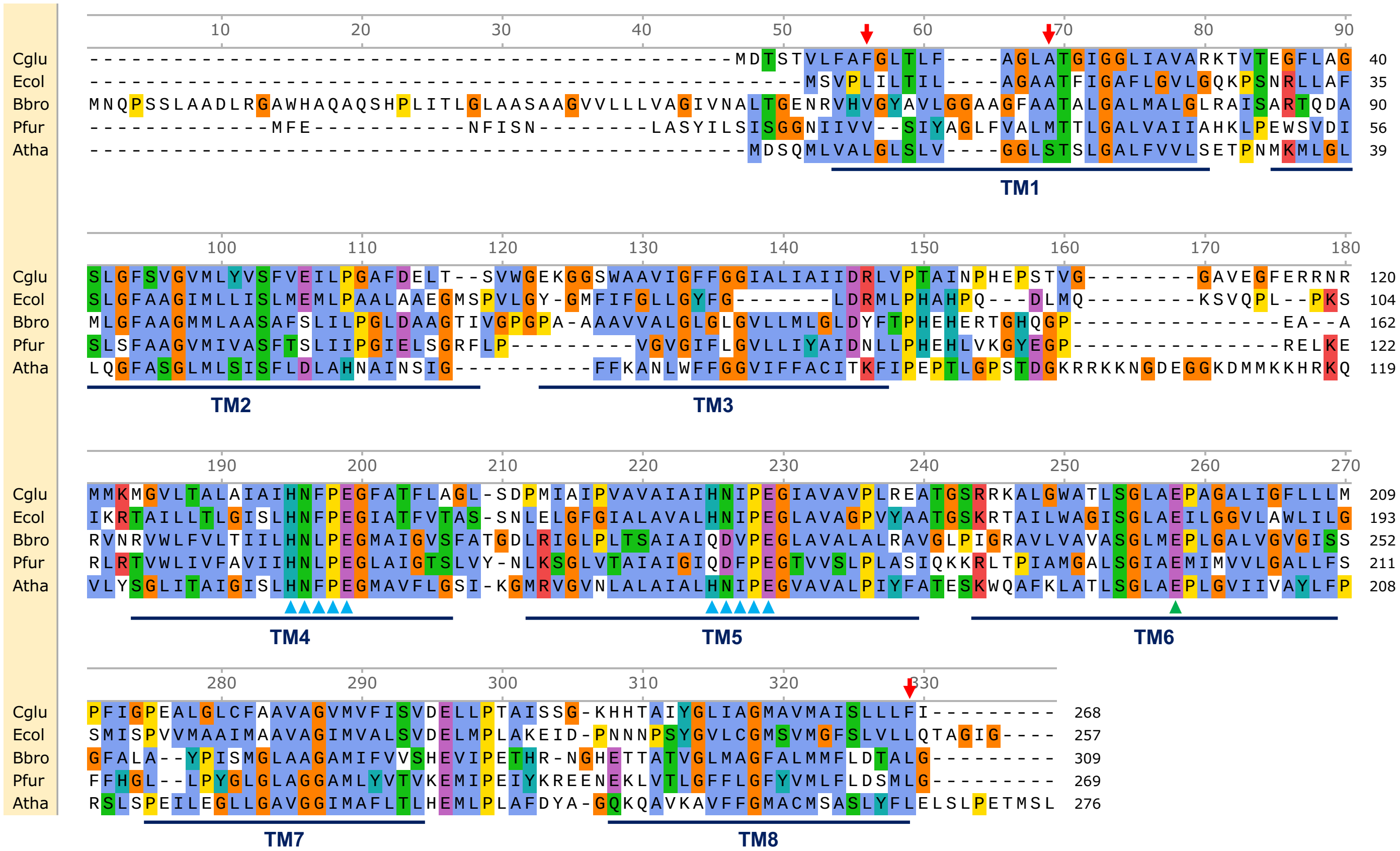

Supplementary Fig. S10 Multiple alignment of amino acid sequences of ZIP family protein homologs from *C. glutamicum* ZupT (NCgl1379) (Cglu), *E. coli* ZupT (Ecol), *Bordetella bronchiseptica* BB2405 (BBro), *Pyrococcus furiosus* PF0746 (Pfur) and *Arabidopsis thaliana* AT3G20870 (Atha). Alignment analysis was performed using CLUSTAL omega (https://www.ebi.ac.uk/jdispatcher/msa/clustalo). The analysis data was visualized with SnapGene Viewer (GSL Biotech LLC). Coloring of amino acid residues is defined following the default Clustal X coloring scheme (https://support.snapgene.com/hc/en-us/articles/10527861802516-What-are-the-SnapGene-Protein-Multiple-Alignment-Color-Highlighting-Schemes). Red arrows represent the amino acid residues where the mutations were found in the cell populations in ALE cultures and the cysteine-resistant mutants of *C. glutamicum*. TM1–TM8 represent eight transmembrane domains of the *B. bronchiseptia* BB2405 determined by its crystal structure (Zhang et al. 2017). Blue and green triangles represent the conserved amino acid residues constituting metal binding domains M1 and M2, respectively.

Supplementary Table S1 Specific growth rate during exponential growth and final OD_{660} value in *C. glutamiucm* wild-type NBRC 12168 strain and its *aecD* gene disruptant.

Strain	Cys·HCl·H_2O concentration (g L^{-1})	Specific growth rate during exponential growth (h^{-1})[a]	Final OD_{660} value[b]
NBRC 12168	No addition	0.23 ± 0.02	3.03 ± 0.15
	0.25	0.21 ± 0.02	1.73 ± 0.18
	0.5	0.16 ± 0.02	1.56 ± 0.03
	1	0.10 ± 0.01	1.69 ± 0.26
aecD gene disruptant	No addition	0.15 ± 0.01	3.07 ± 0.19
	0.25	0.033 ± 0.007	0.22 ± 0.04
	0.5	0.023 ± 0.002	0.22 ± 0.03
	1	0.017 ± 0.006	0.23 ± 0.03

This table was made based on the data in Fig. 1. Average ± standard deviation in three independent culture experiment is shown.

[a]Specific growth rates of the NBRC 12168 strain during exponential growth were calculated using OD_{660} values at 2–8 h. In addition, specific growth rates of the *aecD* gene disruptant were calculated using OD_{660} values at 2–8 h in the absence and presence of 0.25 g L^{-1} Cys·HCl·H_2O and at 3–6 h in the presence of 0.5 and 1 g L^{-1} Cys·HCl·H_2O.

[b]The OD_{660} value at 24 h is shown.

Supplementary Table S2 Summary of genome resequencing analysis of the cell populations in ALE cultures and isolated cysteine-resistant mutants.

Sample	Number of mutations detected	Number of mutations found in ORF	Number of missense mutations in ORF	Number of mutations found in intergenic region between ORFs
aecD disruptant	24	15	10	9
Cell population from ALE2 at 1655.8 h (248 generations)	33	21	13	12
Cell population from ALE2 at 2279 h (362 generations)	39	26	18	13
ALE2_10_3 strain	41	28	18	13
Cell population from ALE5 1680.5 h (240 generations)	34	21	12	13
Cell population from ALE5 at 2279 h (352 generations)	78	56	37	22
ALE5_2_9 strain	103	80	54	23
ALE5_10_4 strain	101	71	48	30

Mutations include single nucleotide variants, insertions and deletions.

Supplementary Table S3 Mutations found in the *zupT* gene in the cell populations of ALE and cysteine-resistant mutants

Cell population of ALE or cysteine-resistant strain	Mutation found in *zupT* locus		
	Position on the genome	Mutation	Corresponding amino acid substitution
Cell population from ALE2 at 1655.8 h (248 generations)	1512228	G→T	Ala19→Ser
Cell population from ALE2 at 2279 h (362 generations)	1512228	G→T	Ala19→Ser
ALE2_10_3 strain	1512228	G→T	Ala19→Ser
Cell population from ALE5 1680.5 h (240 generations)	1512973	T→A	Phe267→Tyr
Cell population from ALE5 at 2279 h (352 generations)	1512196	T→C	Phe8→Ser
	1512973	T→A	Phe267→Tyr
ALE5_2_9 strain	1512196	T→C	Phe8→Ser
ALE5_10_4 strain	1512196	T→C	Phe8→Ser

Supplementary Table S4 Mutations found not in the cell population from ALE2 at 1655.8 h but in the cell population from ALE2 at 2279 h and the ALE2_10_3 strain

Position on the genome	Gene ID	Annotation	Mutation	Corresponding amino acid substitution	Cell population from ALE2 at 1655.8 h	Cell population from ALE2 at 2279 h	ALE2_10_3 strain
860356	NCglr05	23S ribosomal RNA	G→T	None	–	+	+
860357	NCglr05	23S ribosomal RNA	T→C	None	–	+	+
998518	NCgl0906	Putative UDP-N-acetylglucosamine pyrophosphorylase (*glmU*)	C→A	Lys464→Asn	–	–	+
1421742	NCgl1304	Ribosomal protein S1 (*rpsA*)	C→T	Ser215→Phe	–	+	+
2416683	NCgl2203	Undecaprenyl pyrophosphate synthase	C→T	Val140→Met	–	+	+
2577288	NCgl2350	ABC-type transporter, duplicated ATPase component	A→C	Leu529→Pro	–	+	–

"+" and "–" represent presence and absence of the mutation, respectively.

Supplementary Table S5 Mutations found not in the cell population from ALE5 at 1680.5 h but in the cell population from ALE5 at 2279 h and the ALE5_2_9 and ALE5_10_4 strains

Position on the genome	Gene ID	Annotation	Mutation	Corresponding amino acid substitution	Cell population from ALE5 at 1680.5 h	Cell population from ALE5 at 2279 h	ALE5_2_9 strain	ALE5_10_4 strain
38544	NCgl0037	ABC-type transporter, ATPase component	G→A	Glu115→Lys	–	–	–	+
45498	NCgl0043	Bacterial cell division membrane protein (*rodA*)	G→A	Ser391→Leu	–	–	+	–
72221	NCgl0068	Two-component system, response regulators consisting of a CheY-like receiver domain and a HTH DNA-binding domain (*citB*)	G→T	Ala21→Ser	–	+	–	–
87772	NCgl0079	Predicted acetyltransferases and hydrolases with the alpha/beta hydrolase fold	G→A	Thr254→Ile	–	–	–	+
92046	NCgl0085	Urea amidohydrolase (urease) alpha subunit (*ureC*)	G→A	Gly19→Ser	–	–	+	–
137680	NCgl0124	Hypothetical protein	C→T	Pro226→Ser	–	–	–	+
180667	NCgl0164	Hypothetical protein	G→A	Ala327→Thr	–	–	+	–
231097	NCgl0213	ABC-type transporter, ATPase component	C→T	Arg249→Gln	–	–	–	+
251422	NCgl0232	ABC-type multidrug/protein/lipid transport system, ATPase component	C→T	Thr296→Ile	–	–	+	–
257353	NCgl0237	Aspartate transaminase (*aspB*)	G→A	Gly246→Asp	–	–	+	–
355628	NCgl0331	Hypothetical protein	C→T	Arg70→His	–	–	–	+
439786	NCgl0402	Glutamyl-tRNA reductase (*hemA*)	G→A	Gly425→Asp	–	+	+	+
458335	NCgl0419	Cation transport ATPase	C→T	His357→Tyr	–	–	–	+
464216	NCgl0423	Phosphoglycerate mutase/fructose-2,6-bisphosphatase	G→A	Ser117→Asn	–	+	–	–
473264	NCgl0434	Hypothetical protein	C→T	Pro106→Leu	–	+	–	–
486631	NCgl0449	O-succinylbenzoate synthase and related enzyme (*menC*)	G→A	Ala211→Thr	–	–	+	–
599502	NCgl0560	Hypothetical protein	C→T	Pro437→Ser	–	–	+	–
604729	NCgl0566	Predicted hydrolases or acyltransferases (alpha/beta hydrolase superfamily)	G→A	Gly101→Asp	–	–	+	–
686811	NCgl0641	Exonuclease III	G→A	Gly126→Asp	–	+	+	+
697230	NCgl0651	Hypothetical protein	C→T	Gly256→Arg	–	+	+	+
714230	NCgl0664	Uncharacterized protein involved in propionate catabolism (*prpD1*)	T→C	Leu498→Ser	–	–	–	+

733207	NCgl0682	K^+ transporter	C→T	His50→Tyr	–	–	+	–
747114	NCgl0698	ABC-type transporter, ATPase component	G→A	Pro305→Ser	–	–	+	–
803310	NCgl0732	Hypothetical protein	C→T	Val311→Ile	–	–	+	–
861593	NCgl0780	PLP-dependent aminotransferases	T→C	Met1→Thr	–	+	+	+
955953	NCgl0865	FAD/FMN-containing dehydrogenase (*dld*)	G→C	Asp482→Glu	–	+	+	+
962812	NCgl0871	Mg-dependent DNase	T→C	Ser2→Pro	–	+	–	–
979668	NCgl0887	Predicted drug exporter of the RND superfamily	A→G	Leu358→Pro	–	–	–	+
990568	NCgl0896	ABC-type transporter, ATPase component (*urtD*)	C→T	His197→Ser	–	+	+	+
1020899	NCgl0924	Transcription-repair coupling factor - superfamily II helicase (*mfd*)	G→A	Ala609→Thr	–	–	–	+
1028408	NCgl0927	Hypothetical protein	C→T	Gly170→Asp	–	+	+	+
1029082	NCgl0928	Hypothetical protein	C→T	Gly401→Glu	–	–	–	+
1082149	NCgl0986	Na^+-dependent transporters of the SNF family	C→T	Trp171→Stop	–	–	+	–
1089590	NCgl0993	Transposase [*tnp9a* (ISCg9a)]	C→T	Asp51→Asn	–	+	+	+
1117612	NCgl1023	Nicotinate-nucleotide pyrophosphorylase (*nadC*)	C→T	Val45→Met	–	–	+	–
1167327	NCgl1077	Hypothetical protein	G→A	Ser73→Asn	–	+	+	+
1181509	NCgl1087	Shikimate 5-dehydrogenase (*aroE2*)	C→T	Arg56→His	–	–	+	–
1186962	NCgl1092	Permease of the major facilitator superfamily	G→A	Gly380→Ser	–	–	+	–
1219460	NCgl1116	Na^+/proline, Na^+/panthothenate symporters and related permease (*putP*)	G→A	Ala48→Val	–	–	–	+
1226713	NCgl1120	ATPase involved in DNA repair	G→A	Asp550→Asn	–	+	+	+
1273508	NCgl1160	F_0F_1-type ATP synthase c subunit/Archaeal/vacuolar-type H^+-ATPase subunit K (*atpE*)	G→A	Val75→Ile	–	+	+	+
1501791	NCgl1369	Rhodanese-related sulfurtransferase (*sseA2*)	G→A	Val28→Ile	–	–	–	+
1712282	NCgl1552	Predicted nucleoside-diphosphate sugar epimerases (SulA family)	T→C	Val16→Ala	–	–	–	+
1811615	NCgl1647	Hypothetical protein	T→C	Ser18→Pro	–	–	+	–
1837545	NCgl1669	Predicted ATPase (*priP*)	G→A	Asp332→Asn	–	+	+	+
1882838	NCgl1706	Hypothetical protein	A→G	Ser385→Pro	–	–	–	+
1888935	NCgl1711	Hypothetical protein	G→A	Arg54→Cys	–	–	+	–
2034004	NCgl1852	HrpA-like helicase (*hrpA*)	G→A	Glu843→Lys	–	+	+	+
2053042	NCgl1869	tRNA delta(2)-isopentenylpyrophosphate transferase (*miaA*)	G→A	Ala182→Val	–	–	+	–
2136947	NCgl1946	CDP-diglyceride synthetase (*cdsA*)	G→A	Ala48→Val	–	–	–	+
2177275	NCgl1985	Hypothetical protein	C→T	Trp736→Stop	–	–	+	–

2296613	NCgl2088	Hypothetical protein	A→G	Leu96→Pro	–	+	–	–
2311268	NCgl2100	Hypothetical protein	A→G	Asp484→Gly	–	–	+	–
2502226	NCgl2277	Aldo/keto reductases, related to diketogulonate reductase (*dkgA*)	G→A	Pro118→Leu	–	+	+	+
2517323	NCgl2293	Valyl-tRNA synthetase (*valS*)	G→A	Arg342→Cys	–	–	+	–
2613900	NCgl2381	Hypothetical protein	C→T	Pro8→Leu	–	–	–	+
2855938	CNgl2591	Hypothetical protein	G→A	Gly48→Asp	–	–	–	+
2860538	NCgl2594	Lysyl-tRNA synthetase class II (*lysS*)	C→T	Val516→Ile	–	–	+	–
2933583	NCgl2655	Serine/threonine protein kinase (*pknG*)	C→T	His407→Tyr	–	–	+	–
2987482	NCgl2703	Predicted permease	G→A	Asp249→Asn	–	–	+	–
3034378	NCgl2749	Hypothetical protein	C→T	Thr66→Met	–	+	+	+
3089092	NCgl2789	Hypothetical protein	C→T	Ala168→Val	–	–	–	+
3114589	NCgl2811	Hypothetical protein	C→T	Arg324→Cys	–	–	+	–
3188112	NCgl2884	Membrane carboxypeptidase (penicillin-binding protein) (*mrcB*)	G→A	Ala364→Val	–	+	+	+
3222198	NCgl2915	Leucyl-tRNA synthetase (*leuS*)	G→A	Ser146→Phe	–	–	–	+
3231945	NCgl2924	Na^+/H^+-dicarboxylate symporter	G→A	Gln116→Stop	–	+	–	–
3256941	NCgl2951	Protocatechuate 3,4-dioxygenase beta subunit (*catA3*)	A→G	Val145→Ala	–	+	–	–
3263652	NCgl2956	Hypothetical protein	G→A	Glu139→Lys	–	–	–	+
3270585	NCgl2959	Hypothetical protein	C→T	Gln1328→Stop	–	–	–	+

"+" and "–" represent presence and absence of the mutation, respectively.

Reference

Zhang T, Liu J, Fellner M, Zhang C, Sui D, Hu J (2017) Crystal structures of a ZIP zinc transporter reveal a binuclear metal center in the transport pathway. Sci Adv 3:e1700344. doi:10.1126/sciadv.1700344
